# Seroprevalence of Protective Antibodies Against Influenza and the Reduction of the Influenza Incidence Rate: An Annual Repeated Cross‐Sectional Study From 2014 to 2019

**DOI:** 10.1111/irv.13307

**Published:** 2024-05-26

**Authors:** Raquel Guiomar, Susana Pereira da Silva, Inês Costa, Patricia Conde, Paula Cristóvão, Ana Paula Rodrigues, Aida Fernandes, Ana Paula Dias, Ana Rita Couto, Angélica Ramos, Carina Moita, Carina Rodrigues, Fátima Vale, Filomena Caldeira, Jácome Bruges Armas, João Pereira‐Vaz, José Alves, Ludivina Freitas, Luis Martins, Luís Milho, Luisa Mota‐Vieira, Lurdes Lopes, Margarida Freitas, Maria Ana Pessanha, Maria Correia, Maria Helena Marques, Maria João Cardoso, Maria João Peres, Mário Cunha, Patricia Amantegui, Paula Mota, Paulo Lopes, Paulo Pereira, Regina Viseu, Rita Cabral, Rita Côrte‐Real, Sofia Almeida, Vânia Soares, Kamal Mansinho, Olav Hungnes, Baltazar Nunes

**Affiliations:** ^1^ National Reference Laboratory for Influenza and Other Respiratory Viruses, Infectious Diseases Department National Institute of Health Dr. Ricardo Jorge, IP Lisbon Portugal; ^2^ Department of Epidemiology National Institute of Health Dr. Ricardo Jorge, IP Lisbon Portugal; ^3^ Laboratório de Saúde Pública Dr.ª Laura Ayres Faro Portugal; ^4^ Centro Hospitalar de Lisboa Ocidental, E. P. E. Lisbon Portugal; ^5^ Hospital de Santo Espírito da Ilha Terceira, E. P. E.R. Angra do Heroísmo Portugal; ^6^ Centro Hospitalar Universitário de São João, E. P. E. Porto Portugal; ^7^ EPIUnit – Instituto de Saúde Pública Universidade do Porto Porto Portugal; ^8^ Unidade Local de Saúde da Guarda, E. P. E. Guarda Portugal; ^9^ Hospital do Espírito Santo de Évora, E. P. E. Évora Portugal; ^10^ Centro Hospitalar e Universitário de Coimbra, E. P. E. Coimbra Portugal; ^11^ Hospital Central e Universitário da Madeira Funchal Portugal; ^12^ Instituto Português de Oncologia de Lisboa, Francisco Gentil, E.P. E. Lisbon Portugal; ^13^ Hospital do Divino Espirito Santo de Ponta Delgada, E. P. E. R. Ponta Delgada Portugal; ^14^ Centro Hospitalar e Universitário de Lisboa Central, E. P. E. Lisbon Portugal; ^15^ Hospital da Senhora da Oliveira Guimarães, E. P. E. Guimarães Portugal; ^16^ Centro Hospitalar Universitário do Porto, E. P. E. Porto Portugal; ^17^ Centro Hospitalar de Setúbal, E. P. E. Setúbal Portugal; ^18^ Centro Hospitalar Universitário Cova da Beira, E. P. E. Covilhã Portugal; ^19^ Centro Hospitalar de Vila Nova de Gaia/Espinho, E. P. E. Vila Nova de Gaia Portugal; ^20^ Norwegian National Influenza Centre Norwegian Institute of Public Health Oslo Norway

**Keywords:** antibodies, ILI incidence rate, influenza, seroprevalence

## Abstract

**Background:**

Seroepidemiological studies provide estimates of population‐level immunity, prevalence/incidence of infections, and evaluation of vaccination programs. We assessed the seroprevalence of protective antibodies against influenza and evaluated the correlation of seroprevalence with the cumulative annual influenza incidence rate.

**Methods:**

We conducted an annual repeated cross‐sectional seroepidemiological survey, during June–August, from 2014 to 2019, in Portugal. A total of 4326 sera from all age groups, sex, and regions was tested by hemagglutination inhibition assay. Seroprevalence and geometric mean titers (GMT) of protective antibodies against influenza were assessed by age group, sex, and vaccine status (65+ years old). The association between summer annual seroprevalence and the difference of influenza incidence rates between one season and the previous one was measured by Pearson correlation coefficient (*r*).

**Results:**

Significant differences in seroprevalence of protective antibodies against influenza were observed in the population. Higher seroprevalence and GMT for A(H1N1)pdm09 and A(H3N2) were observed in children (5–14); influenza B seroprevalence in adults 65+ was 1.6–4.4 times than in children (0–4). Vaccinated participants (65+) showed significant higher seroprevalence/GMT for influenza. A strong negative and significant correlation was found between seroprevalence and ILI incidence rate for A(H1N1)pdm09 in children between 5 and 14 (*r* = −0.84; 95% CI, −0.98 to −0.07); a weak negative correlation was observed for A(H3N2) and B/Yamagata (*r* ≤ −0.1).

**Conclusions:**

The study provides new insight into the anti‐influenza antibodies seroprevalence measured in summer on the ILI incidence rate in the next season and the need for adjusted preventive health care measures to prevent influenza infection and transmission.

## 1 Introduction

Seroepidemiological studies gained huge relevance during the response to the influenza and COVID‐19 pandemics, helping to assess past infection history, providing estimates of population‐level immunity, estimating the prevalence or incidence of infections (symptomatic and asymptomatic), and evaluating the impact of vaccination programs [1, 2].

The influenza immunity in the population is dynamic, not only due to the influenza (sub)types alternation in circulation in each season but also due to the virus evolution and adaptation. The seroprevalence of protective antibodies against influenza has a changing pattern, thus varying the baseline age‐specific immunity in the population [3]. The contact with the circulating virus and the vaccination boost the population's immunity in each season; however, viral antigenic drift, natural waning immunity, immune imprinting, and immunosenescence are major contributing factors to increasing infection susceptibility [4].

Seroprevalence is an epidemiological tool to understand the annual incidence and to evaluate susceptibility to influenza [5]. Although few national influenza surveillance programs integrate annual serological surveys, these should play an essential role in influenza national and international surveillance systems [6, 7, 8]. The present study aimed to assess the seroprevalence of protective antibodies against influenza virus (naturally acquired or induced after vaccination) and to evaluate the correlation of seroprevalence measured in summer with the influenza like‐illness rate in the following influenza season.

## 2 Methods

In this study, we have conducted an annual repeated cross‐sectional seroepidemiological survey to assess the seroprevalence of protective antibodies against influenza A(H1N1)pdm09, A(H3N2), B/Victoria, and B/Yamagata, from 2014 to 2019, in Portugal's mainland and the Atlantic Islands of Azores and Madeira. Seroprevalence was calculated by age group, sex, vaccine status, and by health administrative region. We also evaluated the correlation between seroprevalence measured in summer and the difference of influenza incidence rates between one season and the previous one for each influenza virus (sub)type.

### 2.1 Sampling

Sixteen laboratories from the Portuguese Laboratory Network for Influenza Diagnosis selected convenience residual sera, according to age and both sexes, between June and August, annually from 2014 to 2019, and provided them irreversibly anonymized to the national reference laboratory.

Individual sera were selected from all age groups (0–4, 5–14, 15–44, 45–64, and 65+ years old), both genders, from all health regions of Portugal: Norte, Centro, Lisboa and Vale do Tejo, Alentejo, Algarve, Azores (São Miguel and Terceira), and Madeira islands. Data on age/date of birth, sex, and district of residence/sample collection were registered; before sera anonymization, the data on previous‐season seasonal influenza vaccine uptake was accessed and recorded for sera from patients 65+ years old (yo), selected from 2017 to 2019. All samples were tested for the detection of antibodies against seasonal influenza viruses by hemagglutination inhibition (HAI) assay [9], using the vaccine strains recommended by the World Health Organization (WHO) for the Northern Hemisphere influenza vaccine, from 2014 to 2019 [10] and new influenza variant viruses, described in the supporting information (Laboratory methods: Serological assay and Table S1).

### 2.2 ILI Incidence

Seasonal ILI incidence rates were estimated for each age group and influenza season using data provided by the Portuguese general practitioners' (GPs') sentinel network that has reported the number of ILI cases on a weekly basis and is the national ILI incidence rate indicator [11]. As numerators were considered all ILI cases that have met ECDC ILI definition [12] with symptoms onset dates falling between start of week 40 of one year and end of week 20 of the following year. The denominator consisted on the average of the population under observation within the GPs' sentinel network during the same periods. Seasonal influenza incidence rates by age group were calculated by multiplying the proportion influenza positive cases (detected by the National Influenza Surveillance Program) by the seasonal ILI incidence rate, to obtain a proxy for seasonal influenza incidence rates by subtype and lineage.

### 2.3 Data Analysis

Seroprevalence estimates were presented with respective 95% confidence intervals (95% CI). Differences between groups (sex, age groups, vaccine status, and region) regarding the proportion of sera with an antibody titer considered protective (titer ≥ 40) were tested using the chi‐square test, while differences in titers between groups were evaluated using the Kruskal–Wallis test. The level of significance was set at 5%. Seroprevalence and geometric mean titer (GMT) ratios were calculated for sex, age group, vaccine status, and region, considering the reference groups: female, 0–4 yo, nonvaccinated, and Norte region, respectively. All statistical analyses were performed in R version 3.0.3. Pearson correlation coefficient (two‐way linear association) for seroprevalence measured in summer and the difference of influenza incidence rates between one season and the previous one was calculated for the overall population and by age group (0–4, 5–14, 15–64, and 65+). Correlation coefficient thresholds were defined as follow: weak (*r* values from 0 to 0.39 or 0 to −0.39), moderate (*r* from 0.40 to 0.59 or −0.40 to −0.59), and strong (*r* from 0.60 to 1 or −0.60 to −1) [13].

### 2.4 Ethics

The sera samples were irreversibly anonymized at each hospital laboratory; the recorded data do not enable the identification of the patient. The present study follows the international ethical guidelines according to the World Medical Association Declaration of Helsinki and was approved by the Health Ethic Committee for Health of the National Institute of Health Dr. Ricardo Jorge (ref. 13/3/2014) and renewed each year, from 2014 to 2019. The study was also approved by the Health Ethic Committee of Hospital of Divino Espírito Santo de Ponta Delgada (ref. 582/CES/2014).

## 3 Results

From 2014 to 2019, 4326 sera were selected from the Portuguese population. The proportions of selected sera were maintained over the 6‐year study. A range of 800 (18.5%) to 901 (20.8%) sera were selected from each age group (0–4, 5–14, 15–44, 45–64, and 65+), and 2181 (51.6%) were from females. From 2017 to 2019, 139 (56.5%) sera from 65+ yo were from vaccinated individuals. Sera selection was carried out, according to the geographic distribution of the population (Table 1).

**TABLE 1:** Demographic characteristics of the sera selected and tested by hemagglutination inhibition (HAI) assay to assess the seroprevalence of protective antibodies against influenza virus between 2014 and 2019.

Serosurvey (year)	Total	2014	2015	2016	2017	2018	2019
Age group	*N* (%)	*N* (%)	*N* (%)	*N* (%)	*N* (%)	*N* (%)	*N* (%)
All ages (*n*)	4326	626	680	708	867	566	879
0–4	800 (18.5%)	119 (19.0%)	128 (18.8%)	128 (18.1%)	151 (17.4%)	113 (20.0%)	161 (18.3%)
5–14	844 (19.5%)	124 (19.8%)	132 (19.4%)	143 (20.2%)	155 (17.9%)	112 (19.8%)	178 (20.3%)
15–44	893 (20.6%)	129 (20.6%)	140 (20.6%)	146 (20.6%)	185 (21.3%)	113 (20.0%)	180 (20.5%)
45–64	901 (20.8%)	129 (20.6%)	140 (20.6%)	147 (20.8%)	188 (21.7%)	117 (20.7%)	180 (20.5%)
65+	888 (20.5%)	125 (20.0%)	140 (20.6%)	144 (20.3%)	188 (21.7%)	111 (19.6%)	180 (20.5%)
Sex^a^							
Female	2181 (51.1%)	311 (49.7%)	343 (50.4%)	358 (50.6%)	447 (51.8%)	278 (53.6%)	444 (50.7%)
Male	2090 (48.9%)	315 (50.3%)	337 (49.6%)	349 (49.4%)	416 (48.2%)	241 (46.4%)	432 (49.3%)
Region							
Norte	1024 (23.7%)	102 (16.3%)	151 (22.2%)	191 (27.0%)	194 (22.4%)	136 (24.0%)	250 (28.4%)
Centro	715 (16.5%)	93 (14.9%)	104 (15.3%)	107 (15.1%)	164 (18.9%)	94 (16.6%)	153 (17.4%)
LVT	1160 (26.8%)	179 (28.6%)	177 (26.0%)	168 (23.7%)	279 (32.2%)	133 (23.5%)	224 (25.5%)
Alentejo	341 (7.9%)	61 (9.7%)	60 (8.8%)	58 (8.2%)	56 (6.5%)	49 (8.7%)	57 (6.5%)
Algarve	241 (5.6%)	41 (6.5%)	39 (5.7%)	43 (6.1%)	36 (4.2%)	35 (6.2%)	47 (5.3%)
Açores	561 (13.0%)	100 (16.0%)	99 (14.6%)	92 (13.0%)	88 (10.1%)	84 (14.8%)	98 (11.1%)
Madeira	284 (6.6%)	50 (8.0%)	50 (7.4%)	49 (6.9%)	50 (5.8%)	35 (6.2%)	50 (5.7%)
Vaccination 65+							
65+ vac	139 (56.5)	▪	▪	▪	54 (66.7%)	33 (58.9%)	52 (47.7%)
65+ nonvac	107 (43.5)	▪	▪	▪	27 (33.3%)	23 (41.1%)	57 (52.3%)

*Note:* ▪ means no data available.

^a^ 55 missing data (1 in 2016, 4 in 2017, 47 in 2018, and 3 in 2019).

### 3.1 Overall Seroprevalence

During the study period, the annual overall seroprevalence for the contemporary vaccine strains of each influenza subtype and lineage varied over time. Seroprevalence and GMT were significantly different for each influenza virus. During 2014, 2015, and 2017, higher seroprevalence rates were observed for influenza A(H3N2). During 2016, 2018, and 2019, higher seroprevalence rates were detected for B/Victoria, B/Yamagata and A(H1N1)pdm09, respectively (Table 2). The GMT reflected the same pattern as the seroprevalence rates (Table S2).

**TABLE 2:** Seroprevalence of protective antibodies (HAI ≥ 40) against influenza A(H1N1)pdm09, A(H3N2), B/Victoria, and B/Yamagata and ratio by age group, between 2014 and 2019, in Portugal (0–4 year old group was considered reference to ratio calculation).

	Age group (*n*)	AH1 seroprev % (*n*) (95% CI)	AH1 ratio (95% CI)	AH3 seroprev % (*n*) (95% CI)	AH3 ratio (95% CI)	B_Victoria seroprev % (*n*) (95% CI)	B_Victoria ratio (95% CI)	B_Yamagata seroprev % (*n*) (95% CI)	B_Yamagata ratio (95% CI)
2014	Overall (626)	26.5 (186) (22.2–31.4)		36.3 (250) (31.2–41.9)		10.4 (57) (7.7–13.6)		27.2 (144) (22.5–32.4)	
	0–4 (119)	33.6 (40) (25.2–42.8)	Ref.	40.3 (48) (31.4–49.7)	Ref.	1.7 (2) (0.2–5.9)	Ref.	7.6 (9) (3.5–13.9)	Ref.
	5–14 (124)	46 (57) (37.0–55.1)	1.4 (1.0–1.9)	59.7 (74) (50.5–68.4)	1.5 (1.1–1.9)	6.5 (8) (2.8–12.3)	3.8 (0.8–17.7)	18.5 (23) (12.1–26.5)	2.5 (1.2–5.1)
	15–44 (129)	24.8 (32) (17.6–33.2)	0.7 (0.5–1.1)	34.9 (45) (26.7–43.8)	0.9 (0.6–1.2)	7 (9) (3.2–12.8)	4.2 (0.9–18.8)	33.3 (43) (25.3–42.2)	4.4 (2.2–8.6)
	45–64 (129)	23.3 (30) (16.3–31.5)	0.7 (0.5–1.0)	24 (31) (16.9–32.3)	0.6 (0.4–0.9)	15.5 (20) (9.7–22.9)	9.2 (2.2–38.6)	20.9 (27) (14.3–29)	2.8 (1.4–5.6)
	65+ (125)	21.6 (27) (14.7–29.8)	0.6 (0.4–1.0)	41.6 (52) (32.9–50.8)	1 (0.8–1.4)	14.4 (18) (8.8–21.8)	8.6 (2.0–36.1)	33.6 (42) (25.4–42.6)	4.4 (2.3–8.7)
2015	Overall (680)	12.1 (85) (9.3–15.3)		20.8 (149) (17–24.9)		8.0 (47) (5.7–10.8)		14.6 (87) (11.4–18.3)	
	0–4 (128)	6.3 (8) (2.7–11.9)	Ref.	21.9 (28) (15.1–30)	Ref.	1.6 (2) (0.2–5.5)	Ref.	4.7 (6) (1.7–9.9)	Ref.
	5–14 (132)	23.5 (31) (16.5–31.6)	3.8 (1.8–7.9)	30.3 (40) (22.6–38.9)	1.4 (0.9–2.1)	4.5 (6) (1.7–9.6)	2.9 (0.6–14.1)	12.1 (16) (7.1–18.9)	2.6 (1–6.4)
	15–44 (140)	10.7 (15) (6.1–17.1)	1.7 (0.8–3.9)	22.1 (31) (15.6–29.9)	1 (0.6–1.6)	7.1 (10) (3.5–12.7)	4.6 (1.0–20.5)	16.4 (23) (10.7–23.6)	3.5 (1.5–8.3)
	45–64 (140)	11.4 (16) (6.7–17.9)	1.8 (0.8–4.1)	13.6 (19) (8.4–20.4)	0.6 (0.4–1.1)	9.3 (13) (5.0–15.4)	5.9 (1.4–25.8)	13.6 (19) (8.4–20.4)	2.9 (1.2–7)
	65+ (140)	10.7 (15) (6.1–17.1)	1.7 (0.8–3.9)	22.1 (31) (15.6–29.9)	1 (0.6–1.6)	11.4 (16) (6.7–17.9)	7.3 (1.7–31.2)	16.4 (23) (10.7–23.6)	3.5 (1.5–8.3)
2016	Overall (708)	36.3 (265) (31.4–41.6)		33.0 (263) (28.5–37.9)		44.3 (323) (39–50.1)		32.0 (222) (27.5–37.1)	
	0–4 (128)	32.8 (42) (24.8–41.7)	Ref.	34.4 (44) (26.2–43.3)	Ref.	39.1 (50) (30.6–48.1)	Ref.	23.4 (30) (16.4–31.7)	Ref.
	5–14 (143)	55.2 (79) (46.7–63.6)	1.7 (1.3–2.2)	62.2 (89) (53.8–70.2)	1.8 (1.4–2.4)	55.2 (79) (46.7–63.6)	1.4 (1.1–1.8)	33.6 (48) (25.9–41.9)	1.4 (1.0–2.1)
	15–44 (146)	37.7 (55) (29.8–46.1)	1.1 (0.8–1.6)	29.5 (43) (22.2–37.6)	0.9 (0.6–1.2)	39 (57) (31.1–47.5)	1 (0.7–1.3)	31.5 (46) (24.1–39.7)	1.3 (0.9–2.0)
	45–64 (147)	33.3 (49) (25.8–41.6)	1 (0.7–1.4)	22.4 (33) (16.0–30.1)	0.7 (0.4–1.0)	43.5 (64) (35.4–52)	1.1 (0.8–1.5)	29.3 (43) (22.0–37.3)	1.2 (0.8–1.9)
	65+ (144)	27.8 (40) (20.6–35.8)	0.8 (0.6–1.2)	37.5 (54) (29.6–45.9)	1.1 (0.8–1.5)	50.7 (73) (42.2–59.1)	1.3 (1.0–1.7)	38.2 (55) (30.2–46.7)	1.6 (1.1–2.4)
2017	Overall (867)	15.0 (138) (12.3–18.0)		35.7 (344) (31.5–40.2)		9.4 (78) (7.3–11.9)		19.7 (159) (16.5–23.4)	
	0–4 (151)	15.9 (24) (10.5–22.7)	Ref.	37.1 (56) (29.4–45.3)	Ref.	4.6 (7) (1.9–9.3)	Ref.	9.9 (15) (5.7–15.9)	Ref.
	5–14 (155)	23.2 (36) (16.8–30.7)	1.5 (0.9–2.3)	72.9 (113) (65.2–79.7)	2 (1.6–2.5)	9.7 (15) (5.5–15.5)	2.1 (0.9–5.0)	18.1 (28) (12.4–25.0)	1.8 (1.0–3.3)
	15–44 (185)	13 (24) (8.5–18.7)	0.8 (0.5–1.4)	31.4 (58) (24.7–38.6)	0.8 (0.6–1.1)	6.5 (12) (3.4–11.1)	1.4 (0.6–3.5)	20 (37) (14.5–26.5)	2.0 (1.1–3.5)
	45–64 (188)	15.4 (29) (10.6–21.4)	1 (0.6–1.6)	25 (47) (19.0–31.8)	0.7 (0.5–0.9)	15.4 (29) (10.6–21.4)	3.3 (1.5–7.4)	20.7 (39) (15.2–27.2)	2.1 (1.2–3.6)
	65+ (188)	13.3 (25) (8.8–19)	0.8 (0.5–1.4)	37.2 (70) (30.3–44.6)	1 (0.8–1.3)	8 (15) (4.5–12.8)	1.7 (0.7–4.1)	21.3 (40) (15.7–27.8)	2.1 (1.2–3.7)
2018	Overall (566)	35.0 (215) (29.7–40.9)		45.8 (287) (39.7–52.4)		32.4 (169) (27.2–38.3)		52.1 (277) (45.3–59.4)	
	0–4 (113)	40.7 (46) (31.6–50.4)	Ref.	50.4 (57) (40.9–60.0)	Ref.	13.3 (15) (7.6–20.9)	Ref.	34.5 (39) (25.8–44.0)	Ref.
	5–14 (112)	53.6 (60) (43.9–63)	1.3 (1.0–1.7)	82.1 (92) (73.8–88.7)	1.6 (1.3–2.0)	34.8 (39) (26.1–44.4)	2.6 (1.5–4.5)	51.8 (58) (42.1–61.3)	1.5 (1.1–2.0)
	15–44 (113)	34.5 (39) (25.8–44)	0.8 (0.6–1.2)	44.2 (50) (34.9–53.9)	0.9 (0.7–1.2)	31 (35) (22.6–40.4)	2.3 (1.4–4.0)	56.6 (64) (47.0–65.9)	1.6 (1.2–2.2)
	45–64 (117)	28.2 (33) (20.3–37.3)	0.7 (0.5–1.0)	33.3 (39) (24.9–42.6)	0.7 (0.5–0.9)	35 (41) (26.5–44.4)	2.6 (1.6–4.5)	46.2 (54) (36.9–55.6)	1.3 (1–1.8)
	65+ (111)	33.3 (37) (24.7–42.9)	0.8 (0.6–1.2)	44.1 (49) (34.7–53.9)	0.9 (0.7–1.2)	35.1 (39) (26.3–44.8)	2.6 (1.6–4.5)	55.9 (62) (46.1–65.3)	1.6 (1.2–2.2)
2019	Overall (879)	49.6 (447) (44.3–55.2)		28.0 (259) (24.2–32.2)		40.9 (337) (36.2–46.1)		32.6 (246) (28.2–37.5)	
	0–4 (161)	50.3 (81) (42.3–58.3)	Ref.	26.7 (43) (20.1–34.2)	Ref.	26.7 (43) (20.1–34.2)	Ref.	10.6 (17) (6.3–16.4)	Ref.
	5–14 (178)	71.3 (127) (64.1–77.9)	1.4 (1.2–1.7)	48.5 (80) (40.6–56.4)	1.8 (1.3–2.5)	31.5 (56) (24.7–38.8)	1.2 (0.8–1.6)	37.1 (66) (30.0–44.6)	3.5 (2.2–5.7)
	15–44 (180)	58.9 (106) (51.3–66.2)	1.2 (1–1.4.0)	26.7 (47) (20.3–33.9)	1 (0.7–1.4)	38.3 (69) (31.2–45.9)	1.4 (1–2)	48.3 (87) (40.8–55.9)	4.6 (2.8–7.4)
	45–64 (180)	34.4 (62) (27.5–41.9)	0.7 (0.5–0.9)	22.2 (40) (16.4–29)	0.8 (0.6–1.2)	46.7 (84) (39.2–54.2)	1.7 (1.3–2.4)	20.6 (37) (14.9–27.2)	1.9 (1.1–3.3)
	65+ (180)	39.4 (71) (32.3–47)	0.8 (0.6–1.0)	27.2 (49) (20.9–34.3)	1 (0.7–1.4)	47.2 (85) (39.8–54.8)	1.8 (1.3–2.4)	21.7 (39) (15.9–28.4)	2.1 (1.2–3.5)

### 3.2 Age Group

A significant difference in seroprevalence and GMT by age group was observed during the study period.

The seroprevalence and the GMT of protective antibodies against influenza A(H1N1)pdm09 and A(H3N2) were higher for the children 5–14 yo. For A(H1N1)pdm09, the highest seroprevalence and the GMT estimates were observed during 2019 summer, 71.3% (64.1–77.9) and 77.2 (62.2–96.0), representing 1.4 (1.2–1.7) and 2.0 (1.4–3.0) times higher than in children 0–4 yo, respectively. For influenza A(H3N2), the highest seroprevalence and the GMT estimates were detected in 2018, 82.1% (73.8–88.7) and 66.9 (55.3–80.9), representing 1.6 (1.3–2.0) and 2.8 (1.7–4.4) times higher than in children 0–4 yo, respectively (Tables 2 and S2).

The lowest seroprevalence of protective antibodies against influenza A viruses was mainly observed in the older age groups (45–64 and 65+ yo). For A(H1N1)pdm09 during summer 2015, the seroprevalence was 10.7% (6.1–17.1) for 65+ yo and 11.4% (6.7–17.9) for 45–64 yo (Table 2). For A(H3N2), the lowest seroprevalences were saw during 2015 summer: 13.6% (8.4–20.4) in adults aged 45–64 yo. The GMT follows the same pattern (Table S2).

For influenza B, the protective antibodies increased with age. Higher seroprevalence was observed in the older age groups, 45–64 yo and 65+ yo. For B/Victoria, higher seroprevalence was estimated during 2016 and 2019 in the group with 65 years or more, after the circulation of this lineage. In these seasons, the seroprevalence of 50.7% in 2016 and 47.2% in 2019 was 1.3 and 1.8 times higher than children under 5 yo, respectively. Exceptionally, during 2016, B/Victoria seroprevalence was also high among the school‐aged children (5–14 yo), being 55.2% (46.7–63.6) (Table 2).

For influenza B/Yamagata, the seroprevalence and GMT were higher for the elderly 65+ yo. Higher seroprevalence and GMT were observed during 2018, 55.9% (46.1–65.3) and 29.1 (24.6–34.4), respectively. The lower seroprevalence and GMT for B/Yamagata were observed in the youngest children, 0–4 yo, during all the study period, with lowest rate measured in summer 2015, 4.7% (1.7–9.9) (Tables 2 and S2).

### 3.3 Gender

The seroprevalence of protective antibodies against influenza A and B and the GMT did not differ significantly between males and females, over the study period (Table S3).

### 3.4 Vaccination Status

The seroprevalence of protective antibodies against influenza and GMT were higher for previous‐season vaccinated compared with the nonvaccinated. Highest seroprevalence and GMT for A(H1N1)pdm09 and A(H3N2) in vaccinated were detected during 2019 and 2017, respectively. GMT in vaccinated was 1.8–2.4 times the GMT measured in the unvaccinated. The same pattern was observed for influenza B seroprevalence and GMT. A 1.2–1.6 times increase was observed in influenza B GMT in vaccinated individuals compared with the unvaccinated (Table 3).

**TABLE 3:** Seroprevalence of protective antibodies (HAI ≥ 40) and geometric means titer (GMT) against influenza A(H1N1)pdm09, A(H3N2), B/Victoria, and B/Yamagata by previous season vaccination status for the target group for influenza vaccine uptake, adults 65+ years old, between 2017 and 2019, in Portugal.

Year			Vaccination status	AH1	Ratio (95% CI)	AH3	Ratio (95% CI)	B_Victoria	Ratio (95% CI)	B_Yamagata	Ratio (95% CI)
2017	Seroprev	% (*n*) (95% CI)	Nvac (*n* = 27)	11.1 (3) (2.4–29.2)	Ref.	29.6 (8) (13.8–50.2)	Ref.	0 0	NA	25.9 (7) (11.1–46.3)	Ref.
			Vac (*n* = 54)	14.8 (8) (6.6–27.1)	1.3 (0.4–4.6)	68.5 (37) (54.4–80.5)	2.3 (1.3–4.3)	13 (7) (5.4–24.9)		18.5 (10) (9.3–31.4)	0.7 (0.3–1.7)
	GMT	(95% CI)	Nvac	7.9 (5.9–10.7)	Ref.	18 (9.7–33.6)	Ref.	8.6 (6.6–11.2)	Ref.	11.7 (8–17.1)	Ref.
			Vac	10.5 (8.0–13.8)	1.3 (0.5–3.3)	43.2 (29.7–62.8)	2.4 (1.4–4.1)	11.4 (8.8–14.6)	1.3 (0.5–3.2)	14.5 (11.5–18.3)	1.2 (0.6–2.7)
2018	Seroprev	% (*n*) (95% CI)	Nvac (*n* = 23)	26.1 (6) (10.2–48.4)	Ref.	39.1 (9) (19.7–61.5)	Ref.	17.4 (4) (5–38.8)	Ref.	52.2 (12) (30.6–73.2)	Ref.
			Vac (*n* = 33)	24.2 (8) (11.1–42.3)	0.9 (0.4–2.3)	48.5 (16) (30.8–66.5)	1.2 (0.7–2.3)	45.5 (15) (28.1–63.6)	2.6 (1.0–6.9)	69.7 (23) (51.3–84.4)	1.3 (0.9–2.1)
	GMT	(95% CI)	Nvac	18.3 (12.1–27.7)	Ref.	25.5 (16.7–38.7)	Ref.	14.8 (11.2–19.6)	Ref.	24.7 (16.1–37.9)	Ref.
			Vac	16.9 (13.4–21.3)	0.9 (0.5–1.8)	29.8 (18.8–47.4)	1.2 (0.7–2.0)	23.2 (17.8–30.2)	1.6 (0.8–1.3)	39.2 (29.5–52.1)	1.6 1.0–2.6)
2019	Seroprev	% (*n*) (95% CI)	Nvac (*n* = 57)	35.1 (20) (22.9–48.9)	Ref.	31.6 (18) (19.9–45.2)	Ref.	43.9 (25) (30.7–57.6)	Ref.	17.5 (10) (8.7–29.9)	Ref.
			Vac (*n* = 52)	55.8 (29) (41.3–69.5)	1.5 (1.0–2.1)	34.6 (18) (22.0–49.1)	1.3 (0.8–2.0)	63.5 (33) (49.0–76.4)	1.2 (0.9–1.5)	26.9 (14) (15.6–41.0)	1.2 (0.7–1.2)
	GMT	(95% CI)	Nvac	24.3 (17–34.8)	Ref.	20.5 (16.5–25.4)	Ref.	20.7 (16.1–26.8)	Ref.	14.8 (11.8–18.5)	Ref.
			Vac	43.3 (29.4–63.8)	1.8	21.1 (16.4–27.2)	1.0	32.3 (24.6–42.5)	1.6	18.2 (14.5–22.9)	1.2

*Note:* Seroprevalence was measured for the upcoming‐season vaccine strains. There was a strain change on 2017 and 2019 for AH1 and 2018 and 2019 for AH3 (nonvaccinated [nvac] group was considered as reference to calculate the seroprevalence and GMT ratios).

Abbreviations: nvac = nonvaccinated (reference); vac = vaccinated.

### 3.5 Regions

We observed a north–south gradient in seroprotection for influenza in each year in Portugal. During 2014, the highest seroprevalence was seen for A(H3N2) at the Norte region 69.4% (59.3–78.3). The lowest seroprevalence was detected at the Alentejo and Algarve, 1.8% (0.0–9.4) for A(H3N2) and 2.6% (0.1–13.5) for B/Yamagata in 2015, respectively. Although the Algarve region lacks sera from less than 5 yo, this pattern remains the same when children under 5 from all regions are excluded from the analysis. Açores and Madeira islands showed similar seroprotection rates to the influenza virus subtypes and B lineages; no significant differences were found. Seroprevalences were also in accordance with GMT values for each virus and region (Tables S4 and S5).

### 3.6 Correlation Between Seroprevalence and Influenza Incidence Rate

We evaluated the correlation between seroprevalence measured in summer and the difference of influenza incidence between one season and the previous one for each influenza A subtype and influenza B lineage, for the overall population, and by age group. For A(H3N2) and B/Victoria, the seroprevalence for the new virus variants detected in circulation during 2014/2015 (A/Switzerland/9715293/2013), 2018 (A/Switzerland/8060/2017), and in 2019 (A/South Australia/34/2019 and B/Washington/02/2019‐like) were considered (Table S6).

#### 3.6.1 A(H1N1)pdm09

A moderate negative correlation (*r* = −0.5; 95% CI, −0.9 to 0.5) between seroprevalence and the influenza incidence rate for A(H1N1)pdm09 was observed among the overall population, 0–4 yo and 65+ yo age groups, indicating that higher seroprevalence was correlated with a decrease in the influenza incidence in next season. A strong negative and significant correlation was found between seroprevalence and influenza incidence rate for A(H1N1) in children between 5 and 14 yo (*r* = −0.8; 95% CI, −1 to −0.1) and in 15–64 yo although nonsignificant (Table 4).

**TABLE 4:** Correlation coefficient (*r*) between seroprevalence measured in summer and the difference of influenza incidence rates between one season and the previous one (ΔILI incidence rate), for each influenza A subtype and influenza B lineage, for overall population and by age group.

Correlation coefficient (*r*) between seroprevalence and ILI incidence rate (95% CI)						
	AH1	AH3	B/Vic	B/Vic^a^ B	B/Yam	B/Yam^a^ B
Overall	−0.5 (−0.9, 0.5)	−0.3 (−0.9, 0.6)	−0.5 (−0.9, 0.5)	−0.3 (−0.9, 0.7)	0.0 (−0.8, 0.8)	−0.1 (−0.8, 0.8)
0–4	−0.3 (−0.9, 0.7)	0.6 (−0.4, 0.9)	−0.1 (−0.8, 0.8)	−0.1 (−0.8, 0.8)	0 (−0.8, 0.8)	−0.2 (−0.9, 0.7)
5–14	−0.8 (−1, −0.1)*	−0.1 (−0.8, 0.8)	−0.5 (−0.9, 0.5)	−0.2 (−0.9, 0.7)	0 (−0.8, 0.8)	−0.1 (−0.8, 0.8)
15–64	−0.7 (−1, 0.3)	−0.3 (−0.9, 0.7)	−0.6 (−0.9, 0.5)	−0.2 (−0.9, 0.7)	0 (−0.8, 0.8)	−0.1 (−0.8, 0.8)
65+	−0.4 (−0.9, 0.6)	−0.2 (−0.9, 0.7)	−0.8 (−1, 0.1)	−0.3 (−0.9, 0.7)	−0.1 (−0.8, 0.8)	−0.1 (−0.8, 0.8)

*Note:* Correlation coefficient thresholds were defined as follow: weak (*r* values from 0 to 0.39 or *r* from 0 to −0.39), moderate (*r* from 0.40 to 0.59 or *r* from −0.40 to −0.59), and strong (*r* from 0.60 to 1 or *r* from −0.60 to −1).

^a^ This was considered the ILI incidence rate for influenza B (both lineages).

* *p* = 0.004.

#### 3.6.2 A(H3N2)

A weak negative correlation between A(H3N2) seroprevalence and influenza incidence rate for the overall population (*r* = −0.3; 95% CI, −0.9 to 0.7) and for individuals aged above 5 yo was found. For children under 5 yo, a strong positive correlation between seroprevalence and the variation of influenza incidence rate was observed (*r* = 0.6; 95% CI, −0.4 to 0.9), contrasting with the overall population and other age groups (Table 4).

#### 3.6.3 B/Victoria and B/Yamagata

For B/Victoria, overall population, a moderate negative correlation (*r* = −0.5; 95% CI, −0.9 to 0.5) was observed; however, a weak negative correlation (*r* = −0.3; 95% CI, −0.9 to 0.7) was seen for influenza B (considering influenza B irrespective of flu B lineage). A strong negative correlation between seroprevalence and influenza incidence rate for B/Victoria was observed for those older than 15 yo (*r* = −0.6; 95% CI, −0.9 to 0.5) and for 65+ (*r* = −0.8; 95% CI, −0.1 to 0.1). This correlation was moderate for 5–14 yo (*r* = −0.5; 95% CI, −0.9 to 0.5) and weak for 0–4 yo (*r* = −0.1; 95% CI, −0.8 to 0.8) (Table 4).

For B/Yamagata, in the overall population and for all age groups, there was a low negative correlation between seroprevalence and influenza incidence rate for B/Yamagata and influenza B (Table 4).

## 4 Discussion

From 2014 to 2019, the annual summer seroprevalence for influenza A subtypes and influenza B lineages varied over time and were significantly different for each tested virus. The seroprevalence and GMT in the overall population for influenza A assumed higher values when compared to influenza B, corresponding well with the viruses that were detected in circulation in the previous influenza seasons.

Due to the evolving nature of the influenza virus, new drift strains with different antigenic and genetic characteristics compared to the vaccine strains were detected in circulation during the study period. Different seroprevalences of protective antibodies against the drift strains were observed, highlighting the existence of cross reactive antibodies to the new circulating strains. Exposure to drifted influenza viruses was able to increase or maintain the pre‐existing immunity [14]. New drift strains not only elicited a monospecific immune response but also promotes a heterologous immunological response for older influenza strains [15].

Significant differences in the seroprevalence of protective antibodies against influenza were observed in the population, mainly according to age, vaccination status, and geographic region. There was no difference in seroprevalence or GMT between sexes over the 6‐year study. This finding is in line with previous studies finding no significant differences in HAI serological response for female and male [16, 17]. The seroprotection rate against influenza A(H1N1)pdm09 and A(H3N2) was higher in children, from 5 to 14 yo, in line with the previous‐season highest ILI incidence rate, especially for the A(H3N2) subtype. These results may suggest the major role of school‐age children in the transmission of influenza in the population [6, 18, 19, 20] and probably reflect the acquisition of antibodies due to previous infection, given that very low levels of vaccine uptake are registered in this age group, under 7% during the study period. In Portugal, for children > 6 months of age, influenza vaccine is only recommended for those with chronic diseases [21, 22].

Although annual influenza vaccination is recommended to the population older than 65 yo, with high vaccine coverage (≥ 60% since 2017/18), the lowest seroprevalence of protective antibodies against influenza A viruses was observed for the older age groups (45–64 and 65+ yo). These results add to existing evidence of immune senescence and pre‐existing immunity and a faster decrease of antibody titers with time and age, more pronounced in the elderly [20, 23]. For influenza B, which only predominated in circulation during one season, 2014/2015, and co‐circulated with A(H3N2) during 2015/2016 and 2017/2018, we observed an increasing trend in the seroprevalence of protective antibodies with age. The 65+ yo showed a higher seroprevalence in the majority of the seasons, even when influenza B was rarely detected. This fact suggests a long‐lasting memory immunity for influenza B in this age group [24]. A previous study also found that the vaccine effectiveness against influenza B declined slightly with time since vaccination, contrary to what was observed for A(H3N2) [25]. Other authors estimated that protection wanes with an average half‐life of 3.5–7 years for children and adults [26]. Children under 4 yo presented the lowest seroprevalence levels of protective antibodies throughout all the study period; these findings are aligned with the estimated age for the first contact with influenza virus infection [27, 28]. It is important to take into account that age seroprevalence is challenging to understand due to the different exposures to a different set of influenza strains resulting from the naturally or vaccine‐accumulated age‐antibody profiles in a population continually exposed to influenza virus [8]. Other study had already demonstrated that pre‐existing immunity affects the rate of seroconversion and immune response due to natural infection or vaccination [14]. Other authors highlighted that a repeated influenza vaccination and natural infections generate complex immune profiles in humans, with a broader antibody landscape in adults than children [29].

During the study period, higher seroprevalence was observed for influenza B/Yamagata than for B/Victoria with the exception in 2016, when B/Victoria had co‐circulated with A(H3N2), and in 2019 after new B/Victoria strain circulation.

A recent study on immune response to influenza vaccination and infection highlighted that the greatest antibody increase postexposure was observed against viruses that circulated within the last 5–8 years, regardless of the priming pattern, vaccine status, or pre‐existing immunity [30]. During the study period and the previous eight seasons, since 2005, the Portuguese population was exposed to four to five seasons dominated by influenza A(H1N1)pdm09 and A(H3N2) virus and one to two B/Victoria and two to three B/Yamagata seasons, consistent with the seroprevalence and GMT pattern that were observed during the study period.

For 65+ yo vaccinated, a clear and significant higher seroprevalence of protective antibodies against influenza A and B viruses and higher antibody titers were seen when compared with unvaccinated aged 65+. These results suggest that influenza vaccination provides effective and consistent antibody levels, and this difference is maintained at least until the subsequent summer. Other studies already showed the clear advantage of vaccine uptake in increasing antibody levels [20]. The data suggest that vaccination against influenza improves seroprevalence and protective antibody titers, contributing to a clearer understanding of the annual recommendations for influenza vaccine uptake, especially for the higher risk groups for severe disease.

Throughout the national territory of Portugal's mainland, a north–south decreasing trend in seroprevalence for protective antibodies against influenza A and B viruses was observed. The reported influenza cases and the seropositivity for influenza in the majority of the seasons follow the same trend [31].

The present study provides a new insight into the relationship between the seroprevalence of protective antibodies against influenza and the reduction of the ILI incidence rate in the next season. These results were more evident for influenza A(H1N1)pdm09 in children between 5 and 14 yo, highlighting the role of the younger population in A(H1N1)pdm09 transmission during the epidemic season, and the importance of acquired immunity in preventing new epidemics. Other studies had highlighted the role of population subgroups in the evolution of epidemics [32] and children played an important role in influenza outbreaks, in household transmission and as a driving force of epidemics in the community [33, 34, 35, 36].

For influenza A(H3N2), due to the virus' higher antigenic and genetic diversity and the lack of previous A(H3N2) infection, the effect of seroprevalence in the reduction of influenza incidence during next season was weak and absent among children under 5 yo.

The results for influenza B/Victoria support the idea that higher seroprevalence will protect and correlate with a lower influenza B incidence in the next season. This was confirmed by the increasing negative correlation trend between seroprevalence and influenza incidence rate with age, with a strong negative correlation for people aged above 15 years old. Although B/Yamagata was detected more frequently and with higher ILI incidence rates during the study period, a low correlation rate was observed between seroprevalence and a reduction in ILI incidence rates, for all ages. In a previous study, it was already observed that influenza B/Yamagata antigens recalled not only a homologous immune response but also a strong booster of B/Victoria antibodies, supporting the cross‐immunity between influenza B lineages [37]. These findings could support the maintenance of the two influenza B lineages in the vaccine composition to broaden the immune response and minimize the chance of B mismatch [10].

Some limitations are recognized in the present study; the sample selection was a nonprobabilistic sample, however, conducted during the inter‐epidemic season and by a wide hospital network covering Portugal's mainland and the Atlantic Islands of Azores and Madeira. The sample collection was performed during summer months, and it is possible that the antibodies previously acquired by vaccination and virus exposure could have waned, although it gives the real picture of the seroprevalence close to the beginning of the next influenza season. Cross‐protection for the new influenza A and B viruses was evaluated for a subsample of sera, nevertheless representing all the study population and covering all age groups and regions. The HAI assay does not assess the levels of all protective antibodies, and cellular immunity was not evaluated, despite having an important role in infection protection. The HAI assay is the “gold‐standard” technique for assessing influenza immunity and measuring antihemagglutinin antibodies guaranteeing the quality, reproducibility, and comparability with other studies. In all assays, reference standards were used. The seroprevalence for influenza B was in line with other studies, even though it was not performed using ether‐treated influenza B antigens, thus prioritizing specificity at the expense of sensitivity and possibly underestimating influenza B seroprevalence. In our study we used HAI ≥ 40 (instead of generally used HI ≥ 80) associated to protection to reduce the possible low sensitivity. Vaccination status from previous‐season vaccine uptake was only collected during 3 years study for the elderly (65+ yo). The vaccine coverage estimates for the 65+ age group in the study sample were very similar to the values estimated for the 65+ Portuguese population with overlapping confidence intervals. We did not observe a higher or lower pattern for the vaccine coverage in the study sample compared to the population. Seroprevalence for all the age groups was calculated with 95% confidence intervals to account for population variability and sampling error.

Estimated seroprevalence is expected to be due to a mixture of natural infection and influenza vaccine uptake. The previous data on ILI incidence rate by age only enabled the analysis of four age categories, resulting in a broad 15–64 age group without allowing the evidence of patterns within subgroups. The correlation analysis only included 6 years study.

## 5 Conclusion

Our study was the first annual seroprevalence survey integrated in the Portuguese influenza surveillance program, shedding light on the importance of the seroprevalence pattern data on predictive models for influenza virus circulation and infection attack rate. Already established serosurveys are also an asset to the pre‐pandemic warning plans and the establishment of pandemic prevention and control measures.

In the future, annual collected serology data could provide estimates of susceptible population, capacitating modelling and epidemiological studies to provide policymakers with important knowledge for planning and preparedness, including guide vaccination plans, and assessment of the likely impact of intervention measures.

## Author Contributions

**Raquel Guiomar:** conceptualization, data curation, investigation, methodology, project administration, resources, supervision, validation, visualization, writing–original draft, writing–review and editing. **Susana Pereira da Silva:** data curation, formal analysis, methodology, validation, writing–review and editing. **Inês Costa:** investigation, methodology, writing–review and editing. **Patricia Conde:** investigation, methodology, writing–review and editing. **Paula Cristóvão:** investigation, methodology, writing–review and editing. **Ana Paula Rodrigues:** investigation, methodology, writing–review and editing. **Aida Fernandes:** investigation, methodology, resources, writing–review and editing. **Ana Paula Dias:** investigation, methodology, resources, writing–review and editing. **Ana Rita Couto:** investigation, methodology, resources, writing–review and editing. **Angélica Ramos:** investigation, methodology, resources, writing–review and editing. **Carina Moita:** investigation, methodology, resources, writing–review and editing. **Carina Rodrigues:** investigation, methodology, resources, writing–review and editing. **Fátima Vale:** investigation, methodology, resources, writing–review and editing. **Filomena Caldeira:** investigation, methodology, resources, writing–review and editing. **Jácome Bruges Armas:** investigation, methodology, resources, writing–review and editing. **João Pereira‐Vaz:** investigation, methodology, resources, writing–review and editing. **José Alves:** investigation, methodology, resources, writing–review and editing. **Ludivina Freitas:** investigation, methodology, resources, writing–review and editing. **Luis Martins:** Investigation, methodology, resources, writing–review and editing. **Luís Milho:** investigation, methodology, resources, writing–review and editing. **Luisa Mota‐Vieira:** investigation, methodology, resources, writing–review and editing. **Lurdes Lopes:** investigation, methodology, resources, writing–review and editing. **Margarida Freitas:** investigation, methodology, resources, writing–review and editing. **Maria Ana Pessanha:** investigation, methodology, resources, writing–review and editing. **Maria Correia:** investigation, methodology, resources, writing–review and editing. **Maria Helena Marques:** investigation, methodology, resources, writing–review and editing. **Maria João Cardoso:** investigation, methodology, resources, writing–review and editing. **Maria João Peres:** investigation, methodology, resources, writing–review and editing. **Mário Cunha:** investigation, methodology, resources, writing–review and editing. **Patricia Amantegui:** investigation, methodology, resources, writing–review and editing. **Paula Mota:** investigation, methodology, resources, writing–review and editing. **Paulo Lopes:** investigation, methodology, resources, writing–review and editing. **Paulo Pereira:** investigation, methodology, resources, writing–review and editing. **Regina Viseu:** investigation, methodology, resources, writing–review and editing. **Rita Cabral:** investigation, methodology, resources, writing–review and editing. **Rita Côrte‐Real:** investigation, methodology, resources, writing–review and editing. **Sofia Almeida:** investigation, methodology, resources, writing–review and editing. **Vânia Soares:** investigation, methodology, resources, writing–review and editing. **Kamal Mansinho:** investigation, methodology, resources, validation, writing–review and editing. **Olav Hungnes:** conceptualization, investigation, methodology, supervision, validation, writing–review and editing. **Baltazar Nunes:** conceptualization, formal analysis, investigation, methodology, supervision, validation, writing–review and editing.

## Conflicts of Interest

The authors declare no conflicts of interest.

### Peer Review

The peer review history for this article is available at https://www.webofscience.com/api/gateway/wos/peer‐review/10.1111/irv.13307.

## Supporting information


**Table S1** Influenza virus strains, A(H1N1), A(H3N2), B/Victoria and B/Yamagata tested in hemagglutination inhibition (HAI) assay in each year between 2014 and 2019. Proportion of detected influenza virus (sub) types in the scope of the Portuguese Influenza Surveillance Program between 2013/2014 and 2018/2019. WHO recommended vaccine strains for the northern Hemisphere influenza vaccine (2013/2014 to 2018/2019).
**Table S2**. Geometric mean titer (GMT) of protective antibodies (HAI ≥ 40) against influenza A(H1N1)pdm09, A(H3N2), B/Victoria and B/Yamagata, and ratio by age group, between 2014 and 2019, in Portugal. (0–4 year old group was considered reference to ratio calculation).
**Table S3**. Seroprotection rates against seasonal influenza by hemagglutination inhibition assay (HI titer≥40), seroprevalence ratio (reference: female) and geometric means titer (GMT) by sex, between 2014 and 2019.
**Table S4**. Seroprevalence rate of protective antibodies (HAI ≥ 40) against influenza A(H1N1)pdm09, A(H3N2), B/Victoria and B/Yamagata, and ratio by health administrative region (Norte, Centro, Lisboa e Vale do Tejo, Alentejo, Algarve, Açores and Madeira), between 2014 and 2019, in Portugal. (Norte region was considered reference for ratio calculation).
**Table S5**. Geometric mean titer (GMT) of protective antibodies (HAI ≥ 40) against influenza A(H1N1)pdm09, A(H3N2), B/Victoria and B/Yamagata, and ratio by health administrative region (Norte, Centro, Lisboa e Vale do Tejo, Alentejo, Algarve, Açores and Madeira), between 2014 and 2019, in Portugal. (Norte region was considered reference for ratio calculation).
**Table S6**. Seroprevalence of protective antibodies (HAI ≥ 40) and GMT for new drifted influenza A(H3N2) and B/Victoria strains, tested on annual serosurvey, between 2014 and 2019.

## Data Availability

The data are publicly available in aggregated format: number of study participants within each level of antibody titer, for each virus strain, each year, each age group, and vaccinated/unvaccinated.
