# Supplementary material for: Seroprevalence of Protective Antibodies Against Influenza and the Reduction of the Influenza Incidence Rate: An Annual Repeated Cross‐Sectional Study From 2014 to 2019
Source: Influenza Other Respir Viruses. 2024 May 26;18(5):e13307. doi: 10.1111/irv.13307 (PMC11128746; doi:10.1111/irv.13307)
Supplement: Supplementary file 1 — Table S1 Influenza virus strains, A(H1N1), A(H3N2), B/Victoria and B/Yamagata tested in hemagglutination inhibition (HAI) assay in each year between 2014 and 2019. Proportion of detected influenza virus (sub) types in the scope of the Portuguese Influenza Surveillance Program between 2013/2014 and 2018/2019. WHO recommended vaccine strains for the northern Hemisphere influenza vaccine (2013/2014 to 2018/2019). Table S2. Geometric mean titer (GMT) of protective antibodies (HAI ≥ 40) against influenza A(H1N1)pdm09, A(H3N2), B/Victoria and B/Yamagata, and ratio by age group, between 2014 and 2019, in Portugal. (0–4 year old group was considered reference to ratio calculation). Table S3. Seroprotection rates against seasonal influenza by hemagglutination inhibition assay (HI titer≥40), seroprevalence ratio (reference: female) and geometric means titer (GMT) by sex, between 2014 and 2019. Table S4. Seroprevalence rate of protective antibodies (HAI ≥ 40) against influenza A(H1N1)pdm09, A(H3N2), B/Victoria and B/Yamagata, and ratio by health administrative region (Norte, Centro, Lisboa e Vale do Tejo, Alentejo, Algarve, Açores and Madeira), between 2014 and 2019, in Portugal. (Norte region was considered reference for ratio calculation). Table S5. Geometric mean titer (GMT) of protective antibodies (HAI ≥ 40) against influenza A(H1N1)pdm09, A(H3N2), B/Victoria and B/Yamagata, and ratio by health administrative region (Norte, Centro, Lisboa e Vale do Tejo, Alentejo, Algarve, Açores and Madeira), between 2014 and 2019, in Portugal. (Norte region was considered reference for ratio calculation). Table S6. Seroprevalence of protective antibodies (HAI ≥ 40) and GMT for new drifted influenza A(H3N2) and B/Victoria strains, tested on annual serosurvey, between 2014 and 2019. [file IRV-18-e13307-s001.docx]

**Supplementary material: Laboratory methods, tables and figure**

**Supplementary table S1. Laboratory methods: Serological assay.**

A minimal remaining volume of 250µl of sera that comes to the laboratory for serological analysis (excluding influenza diagnosis), was refrigerated and sent to the National Reference Laboratory for Influenza and Other Respiratory Viruses at the National Institute of Health Dr. Ricardo Jorge to be tested for the detection of antibodies against seasonal influenza viruses. The selected sera were tested by haemagglutination inhibition (HAI) assay ^14^ using the vaccine strains recommended by the World Health Organization (WHO) for the Northern Hemisphere influenza vaccine, between 2014 and 2019 ^15^, described in Supplementary table S1. During 2014, 2018 and 2019, were also used four A(H3N2) and a B/Victoria influenza strains that were in circulation and were antigenic and genetically different from the vaccine strains (Supplementary table S1). The WHO Collaborating Centre in London kindly provided the influenza reference virus strains and antiserums. Viruses were propagated in Mardin Darbin canine kidney (MDCK) cells and in Mardin Darbin Canine Kidney Sialic Acid Over-Expression cells (MDCK-Siat1) ^14^. All the laboratory work was performed in biosafety level 2 conditions.

Sera were pre-treated with receptor destroying enzyme [RDE (II) ‘‘SEIKEN”; Denka Seiken Co. Ltd.] to remove any unspecific agglutinins and tested in two fold serial dilutions starting at 1:10 to a final dilution of 1:1280, using guinea pig red blood cells. The HAI endpoint titer was assessed as the reciprocal of the highest dilution of serum that completely inhibits haemagglutination. HAI titer ≥ 40 was considered protective against the tested virus strain and for titers <10, 5 were assigned to enable geometric means titer (GMT) calculation. In each assay controls were included to validate and guarantee the quality of the results. The HAI assays had a double blind read by two technicians, and samples with discrepant results (more than 4-fold difference in antibody titer) were re-tested.

**Supplementary table S1**. Influenza virus strains, A(H1N1), A(H3N2), B/Victoria and B/Yamagata tested in heamgglutination inhibition (HAI) assay in each year between 2014 and 2019. Proportion of detected influenza virus (sub) types in the scope of the Portuguese Influenza Surveillance Program between 2013/2014 and 2018/2019. WHO recommended vaccine strains for the northern Hemisphere influenza vaccine (2013/2014 to 2018/2019).

|  |  | HAI Influenza tested virus | | | | Detected Influenza virus (%) | | | | Vaccine Influenza strains | | | | |
| --- | --- | --- | --- | --- | --- | --- | --- | --- | --- | --- | --- | --- | --- | --- |
|  |  | AH1N1 | AH3N2 | B/Victoria | B/Yamagata | AH1N1 | AH3N2 | B/Victoria | B/Yamagata | AH1N1 | AH3N2 | B/Victoria | B/Yamagata |  |
| Season | **Serosurvey** |  |  |  |  |  |  |  |  |  |  |  |  |  |
| 2013/2014 | **2014** | A/California/07/2009 | A/Texas/50/2012 A/H.Kong/5738/2014^¶^ A/Switzerland/9715293/2013^¶^ | B/Brisbane/60/2008 | B/Massachusetts/2/2012 | 60.0 | 40.0 | 0.01 | 0.01 | A/California/7/2009 | A/Victoria/361/2011 | B/Brisbane/60/2008 ^§^ | B/Massachusetts/2/2012 |  |
| 2014/2015 | **2015** | A/California/7/2009 | A/Switzerland/9715293/2013 | B/Brisbane/60/2008 | B/Phuket/3073/2013 | 4.2 | 29.8 | 0.0 | 66.0 | A/California/7/2009 | A/Texas/50/2012 | B/Brisbane/60/2008 ^§^ | B/Massachusetts/2/2012 |  |
| 2015/2016 | **2016** | A/California/7/2009 | A/H.Kong/5738/2014 | B/Brisbane/60/2008 | B/Phuket/3073/2013 | 90.4 | 1.3 | 7.8 | 0.5 | A/California/7/2009 | A/Switzerland/9715293/2013 | B/Brisbane/60/2008 ^§^ | B/Phuket/3073/2013 |  |
| 2016/2017 | **2017** | A/Michigan/45/2015 | A/H.Kong/4801/2014 | B/Brisbane/60/2008 | B/Phuket/3073/2013 | 0.2 | 99.6 | 0.2 | 0.0 | A/California/7/2009 | A/H.Kong/4801/2014 | B/Brisbane/60/2008 | B/Phuket/3073/2013^§^ |  |
| 2017/2018 | **2018** | A/Michigan/45/2015 | A/Singapore/ INFIMH-16-00 19/2016  A/Switzerland/8060/2017^¶^ | B/Colorado/06/2017 | B/Phuket/3073/2013 | 20.0 | 14.0 | 9.0 | 57.0 | A/Michigan/45/2015 | A/H.Kong/4801/2014 | B/Brisbane/60/2008 | B/Phuket/3073/2013^§^ |  |
| 2018/2019 | **2019** | A/Brisbane/02/2018 | A/Kansas/14/2017  A/South Australia/34/2019^¶^ | B/Colorado/06/2017  B/Washington/02/2019^¶^ | B/Phuket/3073/2013 | 33.9 | 65.2 | 0.7 | 0.3 | A/Michigan/45/2015 | A/Singapore/ INFIMH-16-00 19/2016 | B/Colorado/06/2017 | B/Phuket/3073/2013^§^ |  |
| ^¶^ new variants; ^§^ Quadrivalent Influenza Vaccine (QIV) | | | |  |  |  |  |  |  |  |  |  |  |  |

**Supplementary table S2.** Supplementary Material. Geometric mean titer (GMT) of protective antibodies (HAI≥40) against influenza A(H1N1)pdm09, A(H3N2), B/Victoria and B/Yamagata, and ratio by age group, between 2014 and 2019, in Portugal. (0-4 year old group was considered reference to ratio calculation.

|  | Age group  (n) | AH1  GMT  (95% CI) | AH1  Ratio  (95% CI) | AH3  GMT  (95% CI) | AH3  Ratio  (95% CI) | B_Victoria  GMT  (95% CI) | B_Victoria Ratio  (95% CI) | B_Yamagata  GMT  (95% CI) | B_Yamagata Ratio  (95% CI) |
| --- | --- | --- | --- | --- | --- | --- | --- | --- | --- |
| 2014 | **Overall (626)** | 18  (13.9-21.8) |  | 26.1  (16.2-33.7) |  | 9.8  (7.7-11.4) |  | 14.8  (9.3-17.9) |  |
|  | 0-4  (119) | 16.8  (13-21.7) | Ref. | 23.8  (17.8-31.8) | Ref. | 6.8  (6.1-7.5) | Ref. | 7.3  (6.3-8.3) | Ref. |
|  | **5-14**  (124) | 26.3  (21.5-32.2) | 1.6  (0.8-2.9) | 44  (34.7-55.8) | 1.8  (1.1-3) | 9.2  (8.2-10.3) | 1.4  (0.5-3.7) | 12.6  (10.6-15) | 1.7  (0.7-4.3) |
|  | **15-44**  (129) | 14.3  (11.8-17.4) | 0.9  (0.4-1.7) | 17.9  (14.7-21.8) | 0.8  (0.4-1.4) | 9.4  (8.3-10.5) | 1.4  (0.5-3.7) | 19.1  (16-22.8) | 2.6  (1.1-6.2) |
|  | **45-64**  (129) | 13.6  (11.2-16.4) | 0.8  (0.4-1.7) | 14.3  (11.5-17.8) | 0.6  (0.3-1.2) | 11.7  (10.2-13.6) | 1.7  (0.7-4.5) | 14.7  (12.5-17.3) | 2  (0.8-4.9) |
|  | **65+**  (125) | 14.1  (11.8-16.8) | 0.8  (0.4-1.7) | 22.7  (17.7-29.1) | 1  (0.5-1.7) | 11.6  (10.1-13.4) | 1.7  (0.7-4.4) | 17.4  (14.8-20.5) | 2.4  (1-5.7) |
| 2015 | **Overall (680)** | 13.2  (10.8-14.4) |  | 15.0  (12.6-16.3) |  | 9.1  (7.7-10.3) |  | 12.2  (11-12.5) |  |
|  | **0-4** (128) | 10.2  (9-11.5) | Ref. | 15.4  (13.3-17.9) | Ref. | 7.3  (6.6-7.9) | Ref. | 10.8  (9.7-12) | Ref. |
|  | **5-14**  (132) | 16  (14.1-18.3) | 1.6  (0.7-3.5) | 17.1  (14.8-19.8) | 1.1  (0.6-2.2) | 9  (8.1-9.9) | 1.2  (0.5-3.3) | 12.6  (11.1-14.3) | 1.2  (0.5-2.6) |
|  | **15-44**  (140) | 11.5  (10.4-12.7) | 1.1  (0.5-2.6) | 13.5  (11.5-15.9) | 0.9  (0.4-1.8) | 8.3  (7.5-9.2) | 1.1  (0.4-3.1) | 12.5  (11-14.2) | 1.2  (0.5-2.6) |
|  | **45-64**  (140) | 12.1  (10.8-13.7) | 1.2  (0.5-2.7) | 11.5  (10-13.4) | 0.7  (0.3-1.6) | 9.8  (8.8-11) | 1.3  (0.5-3.5) | 10.9  (9.6-12.5) | 1  (0.4-2.4) |
|  | **65+**  (140) | 12.2  (10.9-13.7) | 1.2  (0.5-2.8) | 15.6  (13.2-18.4) | 1  (0.5-2) | 11  (9.7-12.5) | 1.5  (0.6-3.9) | 12.3  (10.8-14) | 1.1  (0.5-2.6) |
| 2016 | **Overall (708)** | 24.8  (18.7-28.9) |  | 25.3  (17.1-31) |  | 25.4  (22.2-27) |  | 20.8  (17.8-22) |  |
|  | **0-4**  (128) | 18.7  (16-22) | Ref. | 20.8  (17.4-24.8) | Ref. | 21.5  (18.6-24.8) | Ref. | 16.7  (14.7-19) | Ref. |
|  | **5-14**  (143) | 34.9  (29.4-41.5) | 1.9  (1.1-3.3) | 38.3  (32.2-45.6) | 1.8  (1.1-3.1) | 28.9  (24.9-33.5) | 1.3  (0.8-2.4) | 21.4  (18.7-24.5) | 1.3  (0.7-2.4) |
|  | **15-44**  (146) | 21.9  (18.7-25.6) | 1.2  (0.6-2.2) | 19.9  (17.2-23.1) | 1  (0.5-1.8) | 22.6  (19.6-26.1) | 1.1  (0.6-1.9) | 20  (17.5-22.8) | 1.2  (0.6-2.3) |
|  | **45-64**  (147) | 18.5  (16.1-21.1) | 1  (0.5-1.9) | 15.3  (13.3-17.5) | 0.7  (0.4-1.4) | 24.2  (21.3-27.3) | 1.1  (0.6-2) | 19.1  (17-21.5) | 1.1  (0.6-2.2) |
|  | **65+**  (144) | 19.1  (16.3-22.3) | 1  (0.5-1.9) | 22  (18.9-25.7) | 1.1  (0.6-1.9) | 24.6  (21.3-28.4) | 1.1  (0.6-2) | 23  (20.3-26.1) | 1.4  (0.7-2.6) |
| 2017 | **Overall (867)** | 9.9  (8.7-10.6) |  | 27.9  (15.8-38.3) |  | 8.8  (7.6-9.8) |  | 11.6  (9.1-12.3) |  |
|  | **0-4**  (151) | 8.5  (7.2-9.9) | Ref. | 19  (15.2-23.9) | Ref. | 7  (6.3-7.8) | Ref. | 8 (7.1-9) | Ref. |
|  | **5-14**  (155) | 11.3  (9.5-13.4) | 1.3  (0.5-3.2) | 57.2  (47.5-68.9) | 3  (1.8-5.1) | 8.6  (7.5-9.7) | 1.2  (0.4-3.3) | 11.4  (10-13.1) | 1.4  (0.6-3.5) |
|  | **15-44**  (185) | 9.2  (8.1-10.5) | 1.1  (0.4-2.8) | 17.5  (14.7-20.7) | 0.9  (0.5-1.8) | 8.5  (7.7-9.5) | 1.2  (0.4-3.3) | 11.9  (10.4-13.6) | 1.5  (0.6-3.6) |
|  | **45-64**  (188) | 9.8  (8.4-11.5) | 1.2  (0.5-2.9) | 14.3  (12.2-16.7) | 0.8  (0.4-1.5) | 10.2  (8.9-11.6) | 1.5  (0.6-3.8) | 11.7  (10.1-13.4) | 1.5  (0.6-3.6) |
|  | **65+**  (188) | 8.9  (7.8-10.2) | 1.1  (0.4-2.7) | 21  (17.3-25.5) | 1.1  (0.6-2.1) | 9.7  (8.7-10.9) | 1.4  (0.5-3.7) | 12.7  (11.2-14.4) | 1.6  (0.7-3.8) |
| 2018 | **Overall (566)** | 23.3  (18.6-26.1) |  | 36  (21-47.6) |  | 17.8  (12.5-20) |  | 28.8  (18.6-30.6) |  |
|  | **0-4**  (113) | 19.4  (15.8-23.9) | Ref. | 24  (19.1-30.3) | Ref. | 10.3  (8.9-12) | Ref. | 15.3  (12.7-18.4) | Ref. |
|  | **5-14**  (112) | 30.5  (25.8-36) | 1.6  (0.9-2.8) | 66.9  (55.3-80.8) | 2.8  (1.7-4.4) | 16.8  (14.3-19.8) | 1.6  (0.8-3.5) | 32  (26.4-38.7) | 2.1  (1.1-3.9) |
|  | **15-44**  (113) | 20.8  (17.4-24.8) | 1.1  (0.6-2) | 25.1  (20.8-30.3) | 1  (0.6-1.8) | 18.1  (15.5-21.2) | 1.8  (0.8-3.8) | 28.9  (24.5-34.1) | 1.9  (1-3.5) |
|  | **45-64**  (117) | 17.9  (15-21.3) | 0.9  (0.5-1.8) | 18.5  (15.2-22.6) | 0.8  (0.4-1.4) | 19.9  (16.8-23.5) | 1.9  (0.9-4.1) | 25.8  (21.5-31) | 1.7  (0.9-3.2) |
|  | **65+**  (111) | 19.6  (16.7-23) | 1  (0.5-1.9) | 28.4  (23-34.9) | 1.2  (0.7-2) | 20.9  (17.9-24.4) | 2  (1-4.3) | 29.1  (24.6-34.4) | 1.9  (1-3.5) |
| 2019 | **Overall (879)** | 43.4  (24.3-58.5) |  | 20  (15.4-23.2) |  | 19.7  (17.3-23) |  | 19.0  (11.6-22.9) |  |
|  | **0-4**  (161) | 37.8  (29.4-48.7) | Ref. | 16.3  (14.2-18.8) | Ref. | 17.1  (15-19.6) | Ref. | 9.7  (8.6-11) | Ref. |
|  | **5-14**  (178) | 77.2  (62.2-96) | 2  (1.4-3) | 28.1  (24.1-32.8) | 1.7  (0.9-3.2) | 16.9  (15.1-19) | 1  (0.5-1.9) | 19.9  (17.4-22.8) | 2.1  (1-4.4) |
|  | **15-44**  (180) | 39.2  (32.3-47.7) | 1  (0.7-1.6) | 15.2  (13.3-17.3) | 0.9  (0.5-1.9) | 20.5  (18-23.3) | 1.2  (0.6-2.3) | 26.3  (22.7-30.4) | 2.7  (1.3-5.7) |
|  | **45-64**  (180) | 22.4  (18.3-27.3) | 0.6  (0.4-1) | 15.5  (13.6-17.8) | 0.9  (0.5-1.9) | 23.2  (20.2-26.6) | 1.4  (0.7-2.5) | 14.1  (12.4-16.1) | 1.5  (0.6-3.3) |
|  | **65+**  (180) | 24.3  (19.9-29.8) | 0.6  (0.4-1.1) | 18.1  (15.7-20.8) | 1.1  (0.6-2.2) | 23.7  (20.4-27.5) | 1.4  (0.7-2.6) | 15.7  (13.8-17.8) | 1.6  (0.7-3.6) |

**Supplementary table S3.** Supplementary material. Seroprotection rates against seasonal influenza by hemagglutination inhibition assay (HI titer≥40), seroprevalence ratio (reference: female) and geometric means titer (GMT) by sex, between 2014 and 2019.

|  |  |  |  | AH1 |  | AH3 |  | B_Victoria |  | B_Yamagata |  |
| --- | --- | --- | --- | --- | --- | --- | --- | --- | --- | --- | --- |
| Year |  |  | **Sex** |  | **Seroprev**  **Ratio (95% CI)** |  | **Seroprev**  **Ratio (95% CI)** |  | **Seroprev**  **Ratio (95% CI)** |  | **Seroprev**  **Ratio (95% CI)** |
| 2014 | **Seroprev** | % (n)  (95% CI) | F (n=311) | 31.5 (98)  (26.4-37) | Ref.  0,9  (0,7-1,1) | 41.8 (130)  (36.3-47.5) | Ref.  0,9  (0,8-1,1) | 10.6 (33)  (7.4-14.6) | Ref.  0,7  (0,4-1,2) | 25.1 (78)  (20.4-30.3) | Ref  0,8  (0,6-1,1) |
|  |  |  | M (n=315) | 27.9 (88)  (23.1-33.2) |  | 38.1  (32.7-43.7) |  | 7.6 (24)  (4.9-11.1) |  | 21 (66)  (16.6-25.9) |  |
|  | **GMT** | (95% CI) | F | 17.4  (15.1-20) | Ref.  0,9 | 22.9  (19.7-26.7) | Ref.  1.0 | 9.7  (8.9-10.5) | Ref.  1.0 | 14  (12.5-15.7) | Ref  0.9 |
|  |  |  | M | 15.5  (13.7-17.6) |  | 22.3  (19.1-26.2) |  | 9.5  (8.8-10.3) |  | 13.3  (11.9-14.8) |  |
| 2015 | **Seroprev** | % (n)  (95% CI) | F (n=343) | 10.8 (37)  (7.7-14.6) | Ref.  1,3  (0,9-2) | 21.9 (75)  (17.6-26.6) | Ref.  1.0  (0,8-1,3) | 6.7 (23)  (4.3-9.9) | Ref.  1,1  (0,6-1,8) | 12.2 (42)  (9-16.2) | Ref  1,1  (0,7-1,6) |
|  |  |  | M (n=337) | 14.2 (48)  (10.7-18.4) |  | 22.0 (74)  (17.7-26.8) |  | 7.1 (166)  (4.6-10.4) |  | 13.4 (45)  (9.9-17.5)) |  |
|  | **GMT** | (95% CI) | F | 12.1  (11.3-13) | Ref.  1.0 | 13.8  (12.6-15.2) | Ref.  1.1 | 8.9  (8.3-9.5) | 1,0 | 11.5  (10.7-12.5) | 1,0 |
|  |  |  | M | 12.4  (11.5-13.5) |  | 15.2  (13.7-16.8) |  | 9.1  (8.5-9.8) |  | 12.1  (11.1-13.1) |  |
| 2016 | **Seroprev** | % (n)  (95% CI) | F (n=358) | 36.3 (130)  (31.3-41.5) | Ref.  1,1 (0,9-1,3) | 36.9 (132)  (31.9-42.1)) | Ref.  1 (0,8-1,2) | 46.4 (166)  (41.1-51.7) | Ref.  1.0 (0,8-1,1) | 29.9 (107)  (25.2-34.9) | Ref  1,1 (0,9-1,4) |
|  |  |  | M (n=349) | 38.7 (135)  (33.5-44) |  | 37.2 (130)  (32.2-42.6) |  | 44.7 (156)  (39.4-50.1) |  | 33.0 (115)  (28-38.2) |  |
|  | **GMT** | (95% CI) | F | 21.7  (19.7-24) | Ref.  1,0 | 22.6  (20.4-25.1) | Ref.  1,0 | 24.1  (22.1-26.3) | Ref.  1,0 | 20.1  (18.6-21.7) | Ref  1,0 |
|  |  |  | M | 22.2  (20-24.7) |  | 21.6  (19.4-24) |  | 24.4  (22.3-26.8) |  | 19.9  (18.3-21.7) |  |
| 2017 | **Seroprev** | % (n)  (95% CI) | F (n=447) | 17.7 (79)  (14.2-21.5) | Ref.  0,8  (0,6-1,1) | 40.5 (181)  (35.9-45.2) | Ref.  1  (0,8-1,1) | 8.9 (40)  (6.5-12) | Ref.  1  (0,7-1,6) | 18.8 (84)  (15.3-22.7) | Ref  1  (0,7-1,3) |
|  |  |  | M (n=416) | 14.2 (59)  (11-17.9) |  | 38.7 (161)  (34-43.6) |  | 9.1 (38)  (6.5-12.3) |  | 18 (75)  (14.5-22.1) |  |
|  | **GMT** | (95% CI) | F | 10.1  (9.2-11.1) | Ref.  0.9 | 22.8  (20.2-25.7) | Ref.  0.9 | 8.9  (8.3-9.6) | Ref.  1.0 | 11.5  (10.6-12.5) | Ref  0.9 |
|  |  |  | M | 8.9  (8.1-9.8) |  | 20.9  (18.3-23.8) |  | 8.7  (8.1-9.4) |  | 10.8  (9.9-11.8) |  |
| 2018 | **Seroprev** | % (n)  (95% CI) | F (n=278) | 37.4 (104) (31.7-43.4) | Ref.  0,9  (0,7-1,1) | 49.6 (138)  (43.6-55.7) | Ref.  1.0  (0,8-1,1) | 31.3 (87)  (25.9-37.1) | Ref.  0,8  (0,6-1,1) | 47.1 (131)  (41.1-53.2) | Ref  1.0  (0,8-1,2) |
|  |  |  | M (n=241) | 33.6 (81)  (27.7-40) |  | 47.3 (114)  (40.9-53.8) |  | 25.3 (61)  (19.9-31.3) |  | 46.1 (111)  (39.6-52.6) |  |
|  | **GMT** | (95% CI) | F | 20.9  (18.6-23.5) | Ref.  0.9 | 27.5  (24-31.5) | Ref.  1.0 | 16.1  (14.5-18) | Ref.  1.0 | 24.8  (22.1-27.9) | Ref  1.0 |
|  |  |  | M | 19.7  (17.4-22.2) |  | 27.1  (23.3-31.4) |  | 16.2  (14.6-17.9) |  | 23.9  (21.1-27.1) |  |
| 2019 | **Seroprev** | % (n)  (95% CI) | F (n=444) | 51.8 (230)  (47-56.5) | Ref.  1  (0,8-1,1) | 30.1 (131)  (25.8-34.7) | Ref.  1  (0,8-1,2) | 41.4 (184)  (36.8-46.2) | Ref.  0,8 (0,7-1) | 30.9 (137)  (26.6-35.4) | Ref  0,8  (0,7-1) |
|  |  |  | M (n=432) | 50 (216)  (45.2-54.8) |  | 29.9 (127)  (25.6-34.5) |  | 35 (151)  (30.5-39.7) |  | 25.2 (109)  (21.2-29.6) |  |
|  | **GMT** | (95% CI) | F | 36  (31.2-41.4) | Ref.  1.0 | 17.8  (16.3-19.6) | Ref.  1.0 | 20.6  (19-22.4) | Ref.  .9 | 17.2  (15.8-18.8) | Ref  0.9 |
|  |  |  | M | 36.4  (31.7-41.9) |  | 18.3 (16.7-20) |  | 19.5  (17.9-21.3) |  | 15.7  (14.3-17.2) |  |

**Supplementary table S4.**  Seroprevalence rate of protective antibodies (HAI≥40) against influenza A(H1N1)pdm09, A(H3N2), B/Victoria and B/Yamagata, and ratio by health administrative region (Norte, Centro, Lisboa e Vale do Tejo, Alentejo, Algarve, Açores and Madeira) , between 2014 and 2019, in Portugal. (Norte region was considered reference for ratio calculation).

|  | Region  (N) | AH1  Seroprev % (n)  (95% CI) | AH1  Ratio  (95% CI) | AH3  Seroprev % (n)  (95% CI) | AH3  Ratio  (95% CI) | B_Victoria Seroprev % (n)  (95% CI) | B_Victoria Ratio  (95% CI) | B_Yamagata Seroprev % (n)  (95% CI) | B_Yamagata Ratio  (95% CI) |
| --- | --- | --- | --- | --- | --- | --- | --- | --- | --- |
| 2014 | **Norte (102** | 41.2 (42)  (31.5-51.4) | Ref. | 67.6 (69) (57.7-76.6) | Ref. | 14.7 (15)  (8.5-23.1) | Ref. | 25.5 (26)  (17.4-35.1) | Ref. |
|  | **Centro**  (93) | 31.2 (29)  (22-41.6) | 0.8  (0.5-1.1) | 32.3 (30) (22.9-42.7) | 0.5  (0.3-0.7) | 5.4 (5)  (1.8-12.1) | 0.4  (0.1-1) | 16.1 (15)  (9.3-25.2) | 0.6  (0.4-1.1) |
|  | **LVT**  (179) | 23.5 (42)  (17.5-30.4) | 0.6  (0.4-0.8) | 31.8 (57) (25.1-39.2) | 0.5  (0.4-0.6) | 7.3 (13)  (3.9-12.1) | 0.5  (0.2-1) | 23.5 (42)  (17.5-30.4) | 0.9  (0.6-1.4) |
|  | **Alentejo**  (61) | 16.4 (10)  (8.2-28.1) | 0.4  (0.2-0.7) | 29.5 (18) (18.5-42.6) | 0.4  (0.3-0.7) | 8.2 (5)  (2.7-18.1) | 0.6  (0.2-1.5) | 11.5 (7)  (4.7-22.2) | 0.5  (0.2-1) |
|  | **Algarve**  (41) | 9.8 (4)  (2.7-23.1) | 0.2  (0.1-0.6) | 17.1 (7)  (7.2-32.1) | 0.3  (0.1-0.5) | 2.4 (1)  (0.1-12.9) | 0.2  (0-1.2) | 12.2 (5)  (4.1-26.2) | 0.5  (0.2-1.2) |
|  | **Azores**  (100) | 41 (41)  (31.3-51.3) | 1  (0.7-1.4) | 44 (44)  (34.1-54.3) | 0.7  (0.5-0.8) | 10 (10)  (4.9-17.6) | 0.7  (0.3-1.4) | 35 (35)  (25.7-45.2) | 1.4  (0.9-2.1) |
|  | **Madeira**  (50) | 36 (18)  (22.9-50.8) | 0.9  (0.6-1.4) | 50 (25)  (35.5-64.5) | 0.7  (0.5-1) | 16 (8)  (7.2-29.1) | 1.1  (0.5-2.4) | 28 (14)  (16.2-42.5) | 1.1  (0.6-1.9) |
| 2015 | **Norte (151)** | 19.2 (29)  (13.3-26.4) | Ref. | 43.7 (66) (35.7-52) | Ref. | 7.9 (12)  (4.2-13.5) | Ref. | 21.2 (32)  (15-28.6) | Ref. |
|  | **Centro**  (104) | 19.2 (20)  (12.2-28.1) | 1  (0.6-1.7) | 16.3 (17)  (9.8-24.9) | 0.4  (0.2-0.6) | 8.7 (9)  (4-15.8) | 1.1  (0.5-2.5) | 20.2 (21)  (13-29.2) | 1  (0.6-1.6) |
|  | **LVT**  (177) | 10.7 (19)  (6.6-16.3) | 0.6  (0.3-1) | 23.2 (41) (17.2-30.1) | 0.5  (0.4-0.7) | 9 (16)  (5.3-14.3) | 1.1  (0.6-2.3) | 14.1 (25)  (9.4-20.1) | 0.7  (0.4-1.1) |
|  | **Alentejo**  (60) | 10 (6)  (3.8-20.5) | 0.5  (0.2-1.2) | 1.7 (1)  (0-8.9) | 0  (0-0.3) | 0 (0)  (0-0) | - | 3.3 (2)  (0.4-11.5) | 0.2  (0-0.6) |
|  | **Algarve**  (39) | 7.7 (3)  (1.6-20.9) | 0.4  (0.1-1.2) | 0 (0)  (0-0) | - | 12.8 (5)  (4.3-27.4) | 1.6  (0.6-4.3) | 2.6 (1) (  0.1-13.5) | 0.1  (0-0.9) |
|  | **Azores**  (99) | 4 (4)  (1.1-10) | 0.2  (0.1-0.6) | 15.2 (15)  (8.7-23.8) | 0.3  (0.2-0.6) | 5.1 (5)  (1.7-11.4) | 0.6  (0.2-1.7) | 1 (1)  (0-5.5) | 0  (0-0.3) |
|  | **Madeira**  (50) | 8 (4)  (2.2-19.2) | 0.4  (0.2-1.1) | 18 (9)  (8.6-31.4) | 0.4  (0.2-0.8) | 0 (0)  (0-0) | - | 10 (5)  (3.3-21.8) | 0.5  (0.2-1.1) |
| 2016 | **Norte (191)** | 40.8 (78)  (33.8-48.2) | Ref. | 53.9 (103) (46.6-61.1) | Ref. | 53.4 (102)  (46.1-60.6) | Ref. | 34.6 (66) (27.8-41.8) | Ref. |
|  | **Centro**  (107) | 32.7 (35)  (24-42.5) | 0.8  (0.6-1.1) | 29 (31)  (20.6-38.5) | 0.5  (0.4-0.7) | 29.9 (32)  (21.4-39.5) | 0.6  (0.4-0.8) | 22.4 (24)  (14.9-31.5) | 0.6  (0.4-1) |
|  | **LVT**  (168) | 31 (52)  (24.1-38.5) | 0.8  (0.6-1) | 29.2 (49) (22.4-36.7) | 0.5  (0.4-0.7) | 34.5 (58)  (27.4-42.2) | 0.6  (0.5-0.8) | 23.8 (40)  (17.6-31) | 0.7  (0.5-1) |
|  | **Alentejo**  (58) | 29.3 (17)  (18.1-42.7) | 0.7  (0.5-1.1) | 31 (18)  (19.5-44.5) | 0.6  (0.4-0.9) | 29.3 (17)  (18.1-42.7) | 0.5  (0.4-0.8) | 25.9 (15)  (15.3-39) | 0.7  (0.5-1.2) |
|  | **Algarve**  (43) | 34.9 (15)  (21-50.9) | 0.9  (0.5-1.3) | 25.6 (11) (13.5-41.2) | 0.5  (0.3-0.8) | 23.3 (10)  (11.8-38.6) | 0.4  (0.2-0.8) | 25.6 (11)  (13.5-41.2) | 0.7  (0.4-1.3) |
|  | **Azores**  (92) | 47.8 (44)  (37.3-58.5) | 1.2  (0.9-1.5) | 31.5 (29) (22.2-42) | 0.6  (0.4-0.8) | 71.7 (66)  (61.4-80.6) | 1.3  (1.1-1.6) | 46.7 (43)  (36.3-57.4) | 1.4  (1-1.8) |
|  | **Madeira**  (49) | 49 (24)  (34.4-63.7) | 1.2  (0.9-1.7) | 44.9 (22) (30.7-59.8) | 0.8  (0.6-1.2) | 77.6 (38)  (63.4-88.2) | 1.5  (1.2-1.8) | 46.9 (23)  (32.5-61.7) | 1.4  (1-1.9) |
| 2017 | **Norte (194)** | 24.7 (48)  (18.8-31.4) | Ref. | 47.9 (93) (40.7-55.2) | Ref. | 14.9 (29)  (10.2-20.8) | Ref. | 29.4 (57)  (23.1-36.3) | Ref. |
|  | **Centro**  (164) | 12.8 (21)  (8.1-18.9) | 0.5  (0.3-0.8) | 39 (64)  (31.5-46.9) | 0.8  (0.6-1) | 8.5 (14)  (4.7-13.9) | 0.6  (0.3-1) | 24.4 (40)  (18-31.7) | 0.8  (0.6-1.2) |
|  | **LVT**  (279) | 18.6 (52)  (14.2-23.7) | 0.8  (0.5-1.1) | 38 (106)  (32.3-44) | 0.8  (0.6-1) | 9.7 (27)  (6.5-13.8) | 0.6  (0.4-1.1) | 14 (39)  (10.1-18.6) | 0.5  (0.3-0.7) |
|  | **Alentejo**  (56) | 3.6 (2)  (0.4-12.3) | 0.1  (0-0.6) | 28.6 (16) (17.3-42.2) | 0.6  (0.4-0.9) | 0 (0)  (0-0 | - | 3.6 (2)  (0.4-12.3) | 0.1  (0-0.5) |
|  | **Algarve**  (36) | 0 (0)  (0-0) | - | 27.8 (10) (14.2-45.2) | 0.6  (0.3-1) | 0 (0)  (0-0 | - | 5.6 (2)  (0.7-18.7) | 0.2 (0-0.7) |
|  | **Azores**  (88) | 15.9 (14)  (9-25.2) | 0.6  (0.4-1.1) | 43.2 (38) (32.7-54.2) | 0.9  (0.7-1.2) | 8 (7)  (3.3-15.7) | 0.5  (0.2-1.2) | 13.6 (12)  (7.2-22.6) | 0.5  (0.3-0.8) |
|  | **Madeira**  (50) | 2 (1)  (0.1-10.6) | 0.1  (0-0.6) | 34 (17)  (21.2-48.8) | 0.7  (0.5-1.1) | 2 (1)  (0.1-10.6) | 0.1  (0-1) | 14 (7)  (5.8-26.7) | 0.5  (0.2-1) |
| 2018 | **Norte (136)** | 41.2 (56)  (32.8-49.9) | Ref. | 58.1 (79) (49.3-66.5) | Ref. | 38.2 (52)  (30-47) | Ref. | 55.9 (76) (47.1-64.4) | Ref. |
|  | **Centro**  (94) | 56.4 (53)  (45.8-66.6) | 1.4  (1-1.8) | 60.6 (57)  (50-70.6) | 1  (0.8-1.3) | 34 (32)  (24.6-44.5) | 0.9  (0.6-1.3) | 52.1 (49)  (41.6-62.5) | 0.9  (0.7-1.2) |
|  | **LVT**  (133) | 34.6 (46)  (26.6-43.3) | 0.8  (0.6-1.1) | 60.9 (81) (52.1-69.2) | 1  (0.9-1.3) | 24.8 (33)  (17.7-33) | 0.6  (0.5-0.9) | 44.4 (59)  (35.8-53.2) | 0.8  (0.6-1) |
|  | **Alentejo**  (49) | 6.1 (3)  (1.3-16.9) | 0.1  (0-0.5) | 30.6 (15) (18.3-45.4) | 0.5  (0.3-0.8) | 16.3 (8)  (7.3-29.7) | 0.4  (0.2-0.8) | 36.7 (18)  (23.4-51.7) | 0.7  (0.4-1) |
|  | **Algarve**  (35) | 34.3 (12)  (19.1-52.2) | 0.8  (0.5-1.4) | 42.9 (15) (26.3-60.6) | 0.7  (0.5-1.1) | 25.7 (9)  (12.5-43.3) | 0.7  (0.4-1.2) | 54.3 (19)  (36.6-71.2) | 1  (0.7-1.4) |
|  | **Azores**  (84) | 39.3 (33)  (28.8-50.5) | 1  (0.7-1.3) | 36.9 (31) (26.6-48.1) | 0.6  (0.5-0.9) | 32.1 (27)  (22.4-43.2) | 0.8  (0.6-1.2) | 48.8 (41)  (37.7-60) | 0.9  (0.7-1.1) |
|  | **Madeira**  (35) | 34.3 (12)  (19.1-52.2) | 0.8  (0.5-1.4) | 25.7 (9)  (12.5-43.3) | 0.4  (0.2-0.8) | 22.9 (8)  (10.4-40.1) | 0.6  (0.3-1.1) | 42.9 (15)  (26.3-60.6) | 0.8  (0.5-1.2) |
| 2019 | **Norte**  **(250)** | 46.8 (117)  (40.5-53.2) | Ref. | 35.2 (82) (29.1-41.7) | Ref. | 40.4 (101)  (34.3-46.8) | Ref. | 32.8 (82) (27-39) | Ref. |
|  | **Centro**  (153) | 51.6 (79)  (43.4-59.8) | 1.1  (0.9-1.4) | 29.4 (45) (22.3-37.3) | 0.8  (0.6-1.1) | 37.3 (57)  (29.6-45.4) | 0.9  (0.7-1.2) | 32 (49)  (24.7-40) | 1  (0.7-1.3) |
|  | **LVT**  (224) | 61.6 (138)  (54.9-68) | 1.3  (1.1-1.6) | 31.7 (71) (25.7-38.2) | 0.9  (0.7-1.2) | 38.4 (86)  (32-45.1) | 1  (0.8-1.2) | 25 (56)  (19.5-31.2) | 0.8  (0.6-1) |
|  | **Alentejo**  (57) | 45.6 (26)  (32.4-59.3) | 1  (0.7-1.3) | 28.1 (16)  (17-41.5) | 0.8  (0.5-1.3) | 21.1 (12)  (11.4-33.9) | 0.5  (0.3-0.9) | 26.3 (15)  (15.5-39.7) | 0.8  (0.5-1.3) |
|  | **Algarve**  (47) | 36.2 (17)  (22.7-51.5) | 0.8  (0.5-1.2) | 12.8 (6)  (4.8-25.7) | 0.4  (0.2-0.8) | 38.3 (18)  (24.5-53.6) | 0.9  (0.6-1.4) | 19.1 (9)  (9.1-33.3) | 0.6  (0.3-1.1) |
|  | **Azores**  (98) | 43.9 (43)  (33.9-54.3) | 0.9  (0.7-1.2) | 32.7 (32) (23.5-42.9) | 0.9  (0.7-1.3) | 53.1 (52)  (42.7-63.2) | 1.3  (1-1.7) | 28.6 (28)  (19.9-38.6) | 0.9  (0.6-1.2) |
|  | **Madeira**  (50) | 54 (27)  (39.3-68.2) | 1.2  (0.9-1.5) | 14 (7)  (5.8-26.7) | 0.4  (0.2-0.8) | 22 (11)  (11.5-36) | 0.5  (0.3-0.9) | 14 (7)  (5.8-26.7) | 0.4  (0.2-0.9) |

**Supplementary table S5.** Geometric mean titer (GMT) of protective antibodies (HAI≥40) against influenza A(H1N1)pdm09, A(H3N2), B/Victoria and B/Yamagata, and ratio by health administrative region (Norte, Centro, Lisboa e Vale do Tejo, Alentejo, Algarve, Açores and Madeira), between 2014 and 2019, in Portugal. (Norte region was considered reference for ratio calculation).

|  | Region  (N) | AH1  GMT  (95% CI) | AH1  Ratio  (95% CI) | AH3  GMT  (95% CI) | AH3  Ratio  (95% CI) | B_Victoria  GMT  (95% CI) | B_Victoria Ratio  (95% CI) | B_Yamagata  GMT  (95% CI) | B_Yamagata Ratio  (95% CI) |
| --- | --- | --- | --- | --- | --- | --- | --- | --- | --- |
| 2014 | **Norte (102)** | 22  (16.9-28.6) | Ref. | 55.4  (42.3-72.6) | Ref. | 11.8  (10.1-13.7) | Ref. | 14.1  (11.6-17.3) | Ref. |
|  | **Centro**  (93) | 15.6  (12.4-19.7) | 0.7  (0.4-1.4) | 19.1  (14.8-24.7) | 0.3  (0.2-0.6) | 8.8  (7.7-10) | 0.7  (0.3-1.8) | 11.2  (9.3-13.5) | 0.8  (0.4-1.7) |
|  | **LVT**  (179) | 14.6  (12.3-17.2) | 0.7  (0.3-1.3) | 16.9  (13.9-20.6) | 0.3  (0.2-0.5) | 9  (8.1-10.1) | 0.8  (0.3-1.8) | 14.1  (12.1-16.4) | 1  (0.5-2.1) |
|  | **Alentejo**  (61) | 10.6  (8.1-13.9) | 0.5  (0.2-1) | 16.5  (12.2-22.3) | 0.3  (0.2-0.5) | 7.9  (6.5-9.5) | 0.7  (0.3-1.6) | 9.7  (7.7-12.1) | 0.7  (0.3-1.5) |
|  | **Algarve**  (41) | 10.3  (7.9-13.5) | 0.5  (0.2-1) | 13.3  (8.7-20.5) | 0.2  (0.1-0.4) | 7.3  (5.9-8.9) | 0.6  (0.2-1.6) | 10.3  (7.7-13.9) | 0.7  (0.3-1.6) |
|  | **Azores**  (100) | 23.5  (18.7-29.4) | 1.1  (0.6-1.9) | 25.7  (18.9-34.8) | 0.5  (0.3-0.7) | 11.1  (9.7-12.7) | 0.9  (0.4-2.1) | 18.2  (15.1-21.8) | 1.3  (0.6-2.6) |
|  | **Madeira**  (50) | 18.7  (13.1-26.6) | 0.8  (0.5-1.6) | 24.6  (17.9-33.8) | 0.4  (0.3-0.7) | 11  (9.1-13.3) | 0.9  (0.4-2.1) | 17.2  (13.2-22.3) | 1.2  (0.6-2.5) |
| 2015 | **Norte (151)** | 13.4  (11.9-15.1) | Ref. | 24  (21-27.5) | Ref. | 9.9  (8.9-10.9) | Ref. | 15.5  (13.7-17.6) | Ref. |
|  | **Centro**  (104) | 15  (12.7-17.8) | 1.1  (0.5-2.3) | 15.8  (13.6-18.5) | 0.7  (0.3-1.2) | 11.9  (10.4-13.6) | 1.2  (0.5-2.8) | 15.3  (13.5-17.4) | 1  (0.5-2) |
|  | **LVT**  (177) | 12.1  (11-13.4) | 0.9  (0.4-2) | 14.7  (12.5-17.3) | 0.6  (0.3-1.2) | 8.1  (7.3-8.9) | 0.8  (0.3-2.1) | 11.7  (10.3-13.2) | 0.7  (0.4-1.6) |
|  | **Alentejo**  (60) | 11.6  (9.7-14) | 0.9  (0.4-1.9) | 6  (5.4-6.7) | 0.3  (0.1-0.6) | 5.7  (5.3-6.1) | 0.6  (0.2-1.6) | 6.8  (5.9-7.9) | 0.4  (0.2-1.1) |
|  | **Algarve**  (39) | 10.7  (8.7-13.2) | 0.8  (0.4-1.8) | 7.4  (6.3-8.7) | 0.3  (0.1-0.7) | 9.1  (7-12) | 0.9  (0.4-2.3) | 7.8  (6.5-9.3) | 0.5  (0.2-1.2) |
|  | **Azores**  (99) | 10.8  (9.8-11.9) | 0.8  (0.4-1.8) | 12.7  (11.1-14.6) | 0.5  (0.3-1) | 9.8  (8.8-10.9) | 1  (0.4-2.4) | 10.8  (9.9-11.8) | 0.7  (0.3-1.5) |
|  | **Madeira**  (50) | 9.7  (8.1-11.7) | 0.7  (0.3-1.7) | 15.2  (12.4-18.5) | 0.6  (0.3-1.2) | 8.2  (7.1-9.5) | 0.8  (0.3-2.1) | 10  (8.3-12.1) | 0.6  (0.3-1.4) |
| 2016 | **Norte (191)** | 24.3  (21.6-27.5) | Ref. | 29.4  (25.5-33.8) |  | 27.7  (24.7-31.2) | Ref. | 21.1  (19.3-23.1) | Ref. |
|  | **Centro**  (107) | 17  (14.3-20.2) | 0.7  (0.4-1.3) | 20.7  (16.9-25.3) | 0.7  (0.4-1.2) | 18.3  (15.6-21.3) | 0.7  (0.4-1.2) | 16.5  (14.2-19.2) | 0.8  (0.4-1.5) |
|  | **LVT**  (168) | 19.5  (16.6-22.9) | 0.8  (0.4-1.5) | 18  (15.5-20.8) | 0.6  (0.3-1.1) | 19.9  (17.5-22.7) | 0.7  (0.4-1.3) | 16.6  (14.7-18.7) | 0.8  (0.4-1.5) |
|  | **Alentejo**  (58) | 19.1  (13.9-26.1) | 0.8  (0.4-1.4) | 19.8  (14.2-27.5) | 0.7  (0.4-1.2) | 21.5  (17.4-26.6) | 0.8  (0.4-1.4) | 20.2  (16.6-24.7) | 1  (0.5-1.8) |
|  | **Algarve**  (43) | 20.3  (16.1-25.6) | 0.8  (0.5-1.5) | 19.7  (15.3-25.3) | 0.7  (0.4-1.2) | 16  (12.7-20) | 0.6  (0.3-1.1) | 16  (12.5-20.3) | 0.8  (0.4-1.4) |
|  | **Azores**  (92) | 28.3  (22.8-35.1) | 1.2  (0.7-2) | 20  (16.6-24) | 0.7  (0.4-1.2) | 36  (30.6-42.3) | 1.3  (0.8-2.1) | 27.9  (23.2-33.5) | 1.3  (0.7-2.3) |
|  | **Madeira**  (49) | 30.1  (23.8-38.2) | 1.2 (0.7-2.1) | 26.5  (21-33.5) | 0.9  (0.5-1.5) | 42.3  (35.7-50.2) | 1.5  (0.9-2.5) | 30.1  (25.4-35.8) | 1.4  (0.8-2.5) |
| 2017 | **Norte (194)** | 12  (10.1-14.2) | Ref. | 29.9  (24.5-36.7) |  | 10.2  (8.9-11.6) | Ref. | 13.6  (11.9-15.6) | Ref. |
|  | **Centro**  (164) | 8.8  (7.6-10.2) | 0.7  (0.3-1.8) | 23.3  (19-28.5) | 0.8  (0.5-1.3) | 9.3  (8.3-10.4) | 0.9  (0.4-2.2) | 13.1  (11.4-15.1) | 1  (0.5-2.1) |
|  | **LVT**  (279) | 10.8  (9.6-12.2) | 0.9  (0.4-2.1) | 20.9  (18.1-24.2) | 0.7  (0.4-1.2) | 9.7  (8.9-10.7) | 1  (0.4-2.3) | 11.2  (10.1-12.4) | 0.8  (0.4-1.8) |
|  | **Alentejo**  (56) | 5.9  (5.3-6.7) | 0.5  (0.2-1.3) | 12.8  (9.5-17.3) | 0.4  (0.2-0.8) | 5.9  (5.4-6.6) | 0.6  (0.2-1.6) | 6.2  (5.4-7.2) | 0.5  (0.2-1.2) |
|  | **Algarve**  (36) | 5.3  (4.9-5.8) | 0.4  (0.2-1.2) | 13.3  (9.1-19.6) | 0.4  (0.2-0.8) | 5.1  (4.9-5.3) | 0.5  (0.2-1.5) | 7.1  (5.7-8.8) | 0.5  (0.2-1.3) |
|  | **Azores**  (88) | 9  (7.5-10.9) | 0.8  (0.3-1.8) | 24  (17.8-32.3) | 0.8  (0.5-1.4) | 8.3  (7.1-9.7) | 0.8  (0.3-2) | 10.9  (9.3-12.8) | 0.8  (0.4-1.8) |
|  | **Madeira**  (50) | 6.5  (5.4-7.9) | 0.5  (0.2-1.4) | 14.5  (10.2-20.8) | 0.5  (0.3-0.9) | 6.3  (5.2-7.6) | 0.6  (0.2-1.7) | 8  (6.2-10.4) | 0.6  (0.2-1.4) |
| 2018 | **Norte (136)** | 23.4  (20.4-26.9) | Ref. | 35.9  (29.8-43.4) |  | 19.8  (17.3-22.7) | Ref. | 29.6  (25.4-34.5) | Ref. |
|  | **Centro**  (94) | 33.5  (28.1-40) | 1.4  (0.8-2.4) | 40.3  (32.7-49.7) | 1.1  (0.7-1.8) | 21.7  (17.9-26.3) | 1.1  (0.6-2) | 29.6  (24.4-35.8) | 1  (0.6-1.7) |
|  | **LVT**  (133) | 21.7  (18.5-25.5) | 0.9  (0.5-1.7) | 35.3  (29-43) | 1  (0.6-1.6) | 14.7  (12.6-17.2) | 0.7  (0.4-1.5) | 24  (20.1-28.7) | 0.8  (0.5-1.4) |
|  | **Alentejo**  (49) | 9.3  (7.7-11.3) | 0.4  (0.2-0.8) | 16.6  (11.6-23.9) | 0.5  (0.3-0.8) | 10.9  (8.7-13.6) | 0.5  (0.3-1.2) | 17.4  (12.6-23.8) | 0.6  (0.3-1.1) |
|  | **Algarve**  (35) | 17.8  (11.9-26.4) | 0.8  (0.4-1.4) | 23.9  (13.9-41.1) | 0.7  (0.4-1.1) | 12.4  (8.8-17.6) | 0.6  (0.3-1.3) | 32.8  (20.4-52.7) | 1.1  (0.7-1.8) |
|  | **Azores**  (84) | 19.5  (15.9-24) | 0.8  (0.5-1.5) | 20  (16.2-24.7) | 0.6  (0.3-1) | 18.3  (15.3-21.7) | 0.9  (0.5-1.7) | 22.4  (18.9-26.6) | 0.8  (0.4-1.3) |
|  | **Madeira**  (35) | 17.4  (11.1-27.2) | 0.7  (0.4-1.4) | 16.4  (11.3-23.8) | 0.5  (0.3-0.8) | 14  (10.5-18.6) | 0.7  (0.4-1.4) | 20.4  (15-27.8) | 0.7  (0.4-1.2) |
| 2019 | **Norte**  **(250)** | 32  (26.6-38.5) | Ref. | 20  (17.5-22.9) |  | 20.6  (18.1-23.3) | Ref. | 19.2  (16.9-21.8) | Ref. |
|  | **Centro**  (153) | 39.1  (30.4-50.3) | 1.2  (0.8-1.9) | 16.5  (14.1-19.2) | 0.8  (0.4-1.6) | 19.3  (16.8-22.2) | 0.9  (0.5-1.7) | 17.9  (15.1-21.1) | 0.9  (0.5-1.8) |
|  | **LVT**  (224) | 52  (42-64.5) | 1.6  (1-2.5) | 19.2  (16.8-21.8) | 1  (0.5-1.8) | 21.3  (19.1-23.8) | 1  (0.6-1.9) | 15.3  (13.6-17.2) | 0.8  (0.4-1.6) |
|  | **Alentejo**  (57) | 24.9  (17.7-34.9) | 0.8  (0.5-1.3) | 14.1  (10.9-18.1) | 0.7  (0.4-1.4) | 13.6  (10.3-17.8) | 0.7  (0.3-1.3) | 14.6  (11.1-19.2) | 0.8  (0.4-1.5) |
|  | **Algarve**  (47) | 26.1  (17.1-39.7) | 0.8  (0.5-1.4) | 14.9  (11.2-19.7) | 0.7  (0.4-1.5) | 20.9  (16.7-26.2) | 1  (0.6-1.9) | 11.9  (9.5-15) | 0.6  (0.3-1.3) |
|  | **Azores**  (98) | 29.5  (23.6-36.9) | 0.9  (0.6-1.5) | 22.7  (19.8-26) | 1.1  (0.6-2.1) | 25.8  (22.2-30) | 1.3  (0.7-2.2) | 17  (14.3-20.2) | 0.9  (0.5-1.7) |
|  | **Madeira**  (50) | 31.2  (21.2-45.7) | 1  (0.6-1.6) | 11.5  (9.3-14.2) | 0.6  (0.3-1.2) | 14.9  (11.6-19.2) | 0.7  (0.4-1.4) | 11.5  (9.2-14.4) | 0.6  (0.3-1.2) |

|  |  | New drifted influenza A(H3N2) and B/Victoria strains | | | | |
| --- | --- | --- | --- | --- | --- | --- |
| Serosurvey year ^a)^ |  | **A/H.Kong/5738/2014_AH3** | **A/Switzerland/9715293/2013_AH3** | **A/Switzerland/8060/2017_AH3** | **B/Washighton/02/2019_B/Victoria** | **A/South Australia/34/2019_AH3** |
| 2014 | Seroprevalence % (n/N)  (95% CI) | 45.6 (62/136)  (37.0-54.3) | 46.6 (68/146)  (38.3-55.0) |  |  |  |
|  | GMT  (value; 95% CI) | 25.5  (22.3-29.3) | 24.1  (20.7-27.9) |  |  |  |
| 2018 | Seroprevalence % (n/N)  (95% CI) |  |  | 19.9 (82/385)  (14.9-25.8) |  |  |
|  | GMT  (value; 95% CI) |  |  | 16.5  (12.3-18.8) |  |  |
| 2019 | Seroprevalence% (n/N)  (95% CI) |  |  |  | 12.7 (112/879)  (10.2-15.6) | 61.6 (543/879)  (55.8-67.8) |
|  | GMT  (value; 95% CI) |  |  |  | 10.6  (9.2-13.8) | 58.9  (30.8-73.7) |
| 1. During 2014, sera from all age groups, with protective antibody titers ≥80 against A/Texas/50/2012 were tested to access the protective antibodies against A/Hong Kong/5738/2014 (3C.2a subclade) and A/Switzerland/9715293/2013 (3C.3a subclade) ; in 2018, a random selection of 385 sera were tested to access the protective antibodies against the A(H3N2) A/Switzerland/8060/2017 strain, from the new 3C.2a2 subclade; In 2019,all 879 sera were tested to access the protective antibodies against B/Washighton/02/2019 (subclade 1A(Δ2) and A/South Australia/34/2019 (clade 3C.2a.1b). | | | | | | |

**Supplementary table S6.** Seroprevalence of protective antibodies (HAI≥40) and GMT for new drifted influenza A(H3N2) and B/Victoria strains, tested on annual serosurvey, between 2014 and 2019.
